# In Situ-Generated, Dispersed Cu Catalysts for the Catalytic Hydrogenolysis of Glycerol

**DOI:** 10.3390/molecules27248778

**Published:** 2022-12-11

**Authors:** Iuliana Porukova, Vadim Samoilov, Dzhamalutdin Ramazanov, Mariia Kniazeva, Anton Maximov

**Affiliations:** A.V. Topchiev Institute of Petrochemical Synthesis, RAS, 29 Leninsky Prospect, 119991 Moscow, Russia

**Keywords:** dispersed copper particles, glycerol, propylene glycol, hydrogenolysis, heterogeneous catalysis

## Abstract

The present study is dedicated to the experimental verification of a concept for the hydrogenolysis of glycerol over in situ-generated Cu dispersed particles (Cu-DP). The Cu-DP were generated by in situ reduction of a precursor salt (Cu(OAc)_2_, CuSO_4_, CuCl_2_) in the presence of KOH and were active in glycerol conversion under hydrogen (T = 200–220 °C, p(H_2_) = 1–4 MPa), where 1,2-propylene glycol (PG) and lactic acid (LA) were detected to be the main products. The influence of the reaction conditions (temperature, hydrogen pressure, reaction time, catalyst-to-feed ratio and the KOH/Cu ratio) on the yields of the products is described. It was shown that the selectivity between the PG and LA could be tuned by changing p(H_2_) or by the KOH amount, i.e., higher yields of LA corresponded to lower p(H_2_) and higher alkalinity of the reaction media. The activity of the in situ-generated Cu-DP was found to be comparable to that of an industrial Cu-Cr_2_O_3_ catalyst. The Cu-DP catalysts were characterized by XRD, XPS, HRTEM and SEM. During the reaction, the catalyst evolved by the sintering and recrystallization of the separate Cu-DP; the crystallite sizes after 1 and 15 h reaction times amounted to 35 and 49 nm, respectively.

## 1. Introduction

The need to reduce environmental degradation and to transition to a low-carbon economy requires the search for new solutions to reduce the consumption of non-renewable carbonaceous raw materials. One such solution is the synthesis of the most important large-tonnage compounds traditionally obtained from raw mineral materials using components derived from renewable sources, i.e., so-called “petrochemical substitutes”. For example, propylene glycol (1,2-propanediol) is a dihydric alcohol of great industrial importance that is currently obtained mainly from fossil-derived propylene oxide. Propylene glycol is used as a polyol in the synthesis of polyurethanes and polyesters, as a solvent and plasticizer, and as a base for technical fluids for various purposes, e.g., antifreezes, hydraulic fluids, water-based heat carriers and deicing fluids for aviation needs [1].

Due to the versatile applications of propylene glycol (PG), the development of technologies for its production from renewable raw materials is of real significance. The most promising renewable raw material for PG production is bioglycerol, the market of which experienced tenfold growth in the 2003–2012 period [2]. At present, it is known that a consortium of Oleon and BASF is producing bio-propylene glycol from bioglycerol [3]. At the same time, studies aimed at improving the technology for obtaining propylene glycol from glycerol continue in two main directions, i.e., the search for new methods of processing crude bioglycerol [4] and the development of improved process catalysts with increased activity, selectivity and stability [4,5,6,7,8].

Traditionally, heterogeneous catalysts based on precious metals (Ru, Rh and Pd [9,10,11,12]) and some transition metals (Co [13], Ni [14,15] and Cu [16,17,18,19,20,21]) have been used for hydrogenation and hydrogenolysis processes. The high cost of precious metals and the low selectivity of catalysts based on them for 1,2-propanediol limit their use in the production of propylene glycol, despite their high activity [22]. The low selectivity of hydrogenolysis is associated with side reactions involving the cleavage of C–C bonds in glycerol with the formation of ethylene glycol, ethanol and methanol, as is typical of both platinum group metals and cobalt and nickel.

In contrast to catalysts based on precious metals, in glycerol hydrogenolysis, copper-containing catalysts demonstrate high selectivity for the hydrogenation of C–O bonds but lower catalytic activity than precious metals. In particular, when using heterogeneous catalysts, such as Cu/Al_2_O_3_ [7,8] and Cu/ZnO [16,23,24], the glycerol conversion rate can be 70%, with propylene glycol selectivity of up to 90%. Other advantages of copper-based catalysts include their relative environmental friendliness and high availability. Due to the high selectivity for 1,2-PG and relative affordability of copper as the main component, copper-containing catalysts are extremely promising for industrial applications.

The main problem of copper-containing catalysts is their low activity. For example, at T = 200 °C and p(H_2_) = 200 psi, propylene glycol was produced on industrial catalysts 5%Pt/C and Raney copper at an equivalent weight catalyst-to-feed ratio, while the mass contents of the active components in these catalysts were 5% and 100%, respectively. Increasing the activity of copper-based catalysts may be achieved in two main ways. The first involves the promotion of copper by a second element. The most effective promotion (which is also used in the manufacture of industrial catalysts) is achieved by adding aluminum [4,18,25], chromium [5,17,26] and zinc oxides [16,18,23,24,27,28] to copper, although examples of adding Ni, Mg [21] or B [4,29] are also known. The second way to increase the activity of copper-based catalysts relies on the fact that their activity is proportional to the copper dispersion (which can be expressed in terms of the specific surface area of copper in the catalyst) [30]. In other words, catalysts with more dispersed copper particles have increased activity. The problem of using highly active heterogeneous catalysts is associated not only with their preparation but also with the configuration of the fixed-bed reactors used. The hydrogenolysis of glycerol is exothermic and, as such, the use of a fixed-bed reactor requires additional technology to control temperature gradients inside the apparatus.

An alternative approach to hydrogenolysis is dispersed-phase catalysis in a slurry reactor. The catalyst is a dispersed phase consisting of solid particles; when the average particle size approaches the sub-nanometer range in a slurry reactor, the diffusion inside the particles practically disappears and the process becomes quasi-homogeneous. To apply this approach, the following factors should be considered:

- The dispersion of catalyst particles affects the reaction rate;
- The thermal effect of the reaction complicates the use of a fixed-bed reactor;
- The catalytic dispersed phase can be formed in situ;
- The reaction medium is able to inhibit the coagulation of the particles;
- The reaction products have a boiling point lower than the raw material and can be separated from the reaction mass by distillation.

**Figure 1:**
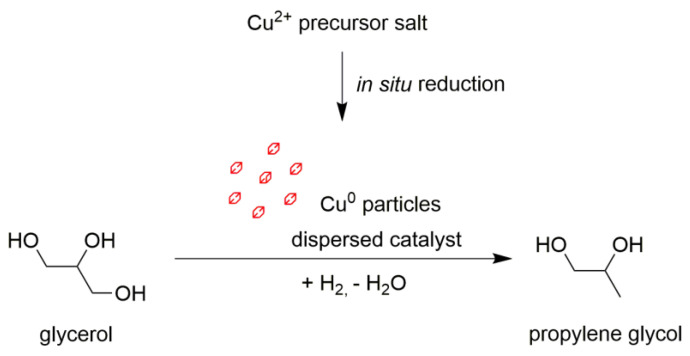
The concept of glycerol hydrogenolysis over in situ-generated ultrafine copper particles.

In the case of the hydrogenolysis reaction of glycerol to propylene glycol, most of these conditions are met. The polyol medium is an excellent stabilizer for dispersed metal particles and has given rise to the polyol process of obtaining dispersions of metal nanoparticles [31]. Copper is easily reduced to a metallic state from Cu^2+^ salts, and polyol itself can act as a reducing agent [32,33,34,35]; the resulting particles can have sizes in the tens of nanometers [36]. This, in turn, led us to consider the application of copper nanoparticles, the activity of which will be high in glycerol hydrogenolysis. The boiling point of propylene glycol is almost 100 °C lower than that of glycerol (190 and 290 °C, respectively), which makes it easy to separate the product by distillation. Finally, the use of a dispersed catalyst in a mixing reactor eliminates the problem of thermal gradients. Ex situ-derived copper nanoparticles were previously successfully applied as a catalyst for furfural hydrogenation [37]. For the hydrogenolysis of glycerol, Omar et al. proposed the use of Cu/ZnO nanoparticles that were also obtained by ex situ synthesis [24]. The aim of this study is to experimentally test our proposed concept for glycerol hydrogenolysis in the presence of in situ-formed, dispersed copper particles (Figure 1).

Achieving the goal requires the following:

- A description of the phenomenon of dispersed catalyst formation in situ in the reaction medium;
- Characterization of the catalytic activity of the resulting catalyst, including the dependence of product yields on the reaction conditions;
- Description of the structure and morphology of in situ-generated catalysts.

## 2. Results and Discussion

### 2.1. Phenomenon of Catalytic Activity in the Glycerol Hydrogenolysis Reaction

The initial hypothesis of our study implies that when a copper salt solution (Figure S5) is heated in glycerol, dispersed particles of metallic copper are formed, which, due to their catalytic activity, catalyze the glycerol hydrogenolysis reaction. Confirmation of this hypothesis should be based on the following observations:

- A heterogeneous catalyst must be present in the reaction mixture;
- The resulting reaction mixture must contain reaction products—in particular, the target PG;
- The conversion of glycerol and the yield of the desired products should increase with increasing temperature and reaction time;
- The conversion of glycerol and the yield of the desired products should change when the Gly/Cu ratio changes;
- Propylene glycol should be formed only in a hydrogen medium and only in the presence of a copper precursor salt.

The activity of copper-containing catalysts in glycerol hydrogenolysis is traditionally considered to be relatively low compared with that of catalysts based on platinum group metals. The results of experiments on glycerol hydrogenolysis using an industrial copper chromite catalyst (Table 1, entry 23–24) have generally confirmed this statement; at a ratio of Gly/Cu = 100 mol and T = 200 °C, the glycerol conversion for 5–10 h of reaction was 3.6–9.2%. Thus, to obtain the measured dynamics in the experiment, the catalyst must be 1–2% mol per substrate.

At this ratio, the precursor cannot be quickly dissolved in the initial water–glycerol mixture; therefore, to ensure homogenization, an excess of KOH was added to the solution with the precursor. In this case, the copper salts were completely dissolved, probably forming copper glycerate, as indicated by the cornflower-blue color of the solution.

When heating precursor solutions in glycerol at T = 200–220 °C and p(H_2_) = 3 MPa, solution discoloration was observed with the simultaneous formation of a copper-colored solid phase. The analysis of reaction mixtures (Table 1) showed that these phenomena were accompanied by glycerol conversion with the formation of two main products, i.e., propylene glycol and lactic acid (or salt, depending on the pH of the reaction medium), and two by-products, i.e., ethylene glycol and glyceric acid (or salt, depending on the pH of the reaction medium). This was observed for all three tested precursors (CuSO_4_, Cu(OAc)_2_ and CuCl_2_). With this in mind, the conversion of glycerol sharply decreased in the absence of a copper salt precursor, and propylene glycol was practically not formed at all. Thus, when heating glycerol with KOH at T = 220 °C (Table 1, entry 1), the glycerol conversion was 2.2%, of which, 2.0% accounted for Y_LA_ and only 0.2% for Y_PG_. The same mixture with the addition of 2% mol (for glycerol) of copper acetate (Table 1, entry 14) was characterized by X_Gly_ = 16.8% with Y_PG_ = 8.5% and Y_LA_ = 6.8%. Thus, the formation of propylene glycol was associated with the presence of copper in the reaction mixture. It was also found that propylene glycol did not form when the reaction took place in a nitrogen atmosphere (Table 1, entry 18–20); the Y_PG_ under these conditions was only 0.7%. When the reaction was carried out under the same conditions in a hydrogen atmosphere (p(H_2_) = 3.0 MPa), the PG yield was 4.0% (Table 1, entry 16). These results confirm that a heterogeneous catalyst was formed from the precursor salt in the solution and that a catalytic reaction of hydrogenolysis subsequently occurred.

The conversion rate of glycerol and the yield of products varied depending on the reaction time and the catalyst-to-feed ratio. For example, in the presence of copper acetate at T = 200 and 220 °C (Table 1, entries 11 and 15), the X_Gly_ amounted to 15.5% and 18.5%, respectively. With a twofold increase in the catalyst-to-feed ratio, glycerol conversion also increased. For instance, at Gly/Cu = 100 mol and 50 mol, the X_Gly_ was 7.8% and 16.8%, respectively (Table 1, entries 16 and 14).

It is obvious that it is the alkaline nature of the reaction mixture that is responsible for the significant yield of lactic acid during the glycerol hydrogenolysis in the presence of in situ-formed copper particles. The co-catalytic performance of KOH was confirmed by a control test. The glycerol hydrogenolysis over the Cu-Cr_2_O_3_ catalyst resulted in X_Gly_ = 19.0%, Y_PG_ = 17.3% and Y_LA_ = 1.5% (Table 1, entry 25). When the fivefold molar amount of KOH was added to the Cu-Cr_2_O_3_ catalyst, the Y_PG_ was slightly increased, while the Y_LA_ rose from 1.5% to 9.4%, therefore contributing to the X_Gly_ surplus. Since the alkali acting as a co-catalyst regulates the reaction selectivity, the corresponding dependence deserves consideration.

Cu^2+^ reduction should be accompanied by partial neutralization of alkali according to the following equations:

Cu(OAc)_2_ + H_2_ = Cu^0^ + 2HOAc; 2HOAC + 2KOH = 2KOAc + 2H_2_O

**Figure 2:**
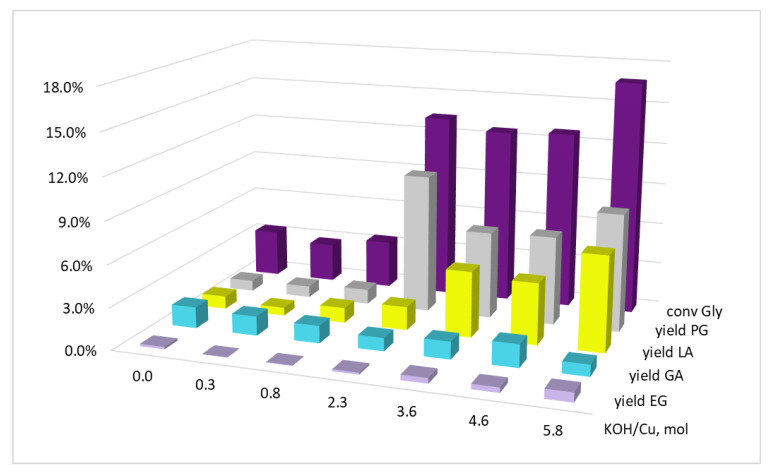
The influence of the KOH/Cu molar ratio on the glycerol conversion and the yields of the products. Conditions: T = 220 °C, p(H_2_) = 3.0 MPa, Gly/Cu = 50 mol, τ = 5 h, precursor salt = Cu(OAc)_2_.

Thus, at the ratio KOH/Cu = 2.0 mol, complete alkali neutralization should be observed during complete copper reduction. At lower KOH/Cu ratios in the initial reaction mixture, one can expect acidic medium formation; the values of KOH/Cu > 2.0 mol will correspond to the alkaline medium. As can be seen from the results obtained (Figure 2), the glycerol conversion and yields of products depended on the alkali amount in the initial reaction mixture. With a lack of alkali relative to stoichiometric amount (KOH/Cu < 2.0 mol), X_Gly_ did not exceed 3.5%, with a Y_PG_ of 0.8–1.1% and Y_LA_ of 0.6–1.1%. When the medium changed to a slightly alkaline one (KOH/Cu = 2.3 mol), the glycerol conversion increased sharply to 13.4%, and this increase was associated with an increase in the propylene glycol yield of up to 10.0%, while the lactic acid yield increased insignificantly (up to 1.7%). A further increase in the alkali amount and the medium transition to alkaline (KOH/Cu = 3.6–5.8 mol) changed the conversion selectivity at an almost constant conversion. At KOH/Cu = 3.6 mol, the glycerol conversion was 12.6%; furthermore, Y_PG_ = 6.3% and Y_LA_ = 4.7%. By-product (EG and GA) yields did not depend much on the alkali amount in the mixture. Thus, the highest selectivity of glycerol conversion in the presence of the in situ-formed copper catalyst was achieved in a slightly alkaline medium; when the dosage was increased, PG and LA alkalis were formed in comparable amounts.

**Figure 3:**
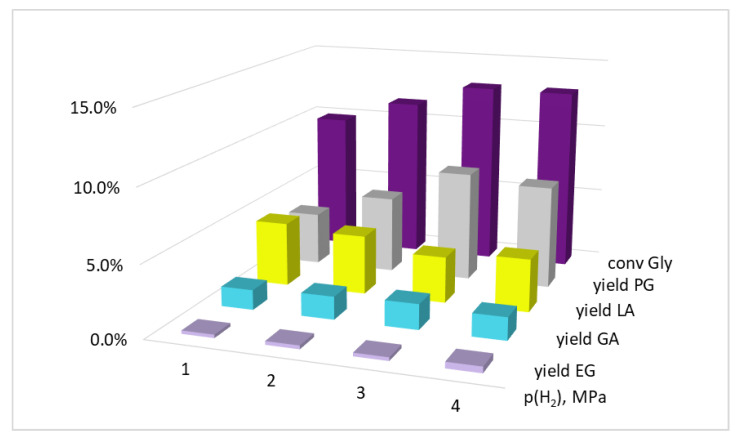
The influence of the hydrogen pressure on the glycerol conversion and the yields of the products. Conditions: T = 220 °C, KOH/Cu = 5.8 mol, Gly/Cu = 50 mol, Gly/H_2_O = 4.1 vol, τ = 2.5 h, precursor salt = Cu(OAc)_2_.

Lactic acid is formed from glycerol in an alkaline medium through successive stages of dehydrogenation (catalyzed by Cu^0^) and dehydration (catalyzed by OH^−^). Both the rate and the equilibrium of the dehydrogenation reaction can depend on the hydrogen pressure in the system. However, the propylene glycol (hydrogenolysis product) formation rate can also depend on the hydrogen pressure. Thus, changing p(H_2_) can be a way to control the reaction selectivity. The results obtained (Figure 3) indicate the validity of these assumptions: an increase in hydrogen pressure was accompanied by both an increase in glycerol conversion and an increase in selectivity between PG and LA. Thus, during glycerol hydrogenolysis in the presence of Cu(OAc)_2_ (T = 220 °C, KOH/Cu = 5.8 mol, Gly/Cu = 50 mol, τ = 2.5 h), at p(H_2_) = 1.0 MPa and 4.0 MPa, X_Gly_ amounted to 9.9% and 13.0%; Y_PG_ amounted to 3.7% and 7.2%; and Y_LA_ decreased from 4.5% to 3.7%, respectively. With that, we did not find a significant effect of hydrogen pressure on the yields of by-products of ethylene glycol and glyceric acid. Despite the lack of data on the reaction kinetics, it can be concluded from this that the glycerol hydrogenolysis reaction with the propylene glycol formation in the presence of the in situ-formed copper dispersed catalysts has a non-zero hydrogen order.

**Figure 4:**
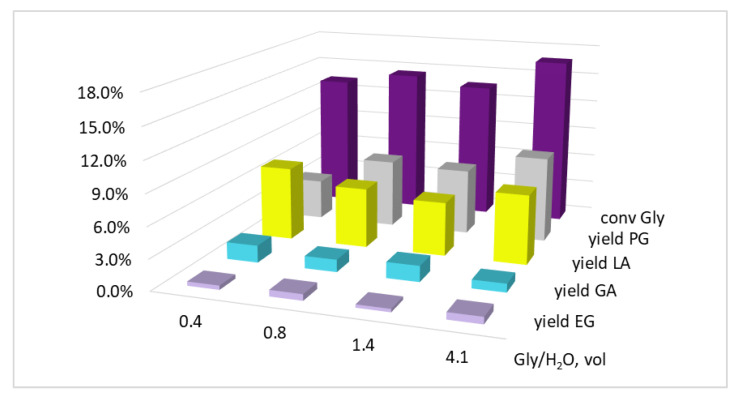
The influence of the Gly/H_2_O volume ratio on the glycerol conversion and the yields of the products. Conditions: T = 220 °C, p(H_2_) = 3.0 MPa, KOH/Cu = 5.8 mol, Gly/Cu = 50 mol, τ = 5.0 h, precursor salt = Cu(OAc)_2_.

With that, both the conversion and the selectivity of the reaction changed little when the glycerol concentration in the reaction mixture was reduced by diluting it with water (Figure 4). Therefore, at Gly/H_2_O = 0.4 vol, the values of X_Gly_/Y_PG_/Y_LA_ were 13.3/4.0/7.3%, and at Gly/H_2_O = 4.1 vol the values were 16.8/8.5/6.8%, respectively. Apparently, the lactic acid formation reaction has an glycerol order close to zero. Since Y_PG_ more than doubled (from 4.0 to 8.5%) with an increase in the glycerol concentration, the propylene glycol formation reaction order obviously has a non-zero value.

**Figure 5:**
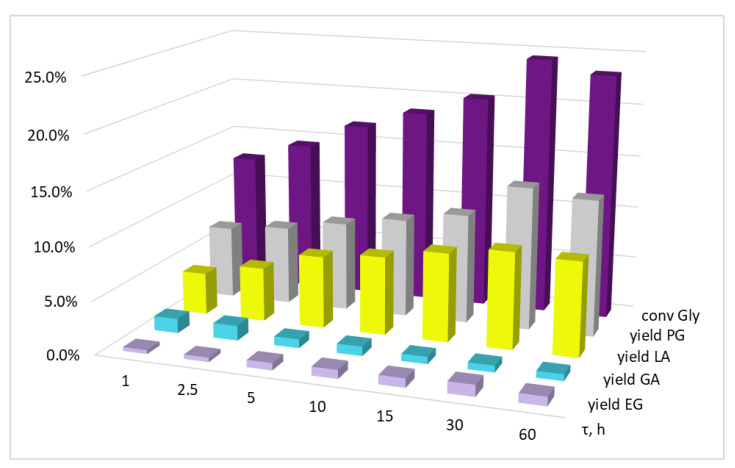
The influence of the reaction time on glycerol conversion and the yields of the products. Conditions: T = 220 °C, p(H_2_) = 3.0 MPa, KOH/Cu = 5.8 mol, Gly/Cu = 50 mol, Gly/H_2_O = 4.1 vol, precursor salt = Cu(OAc)_2_.

**Figure 6:**
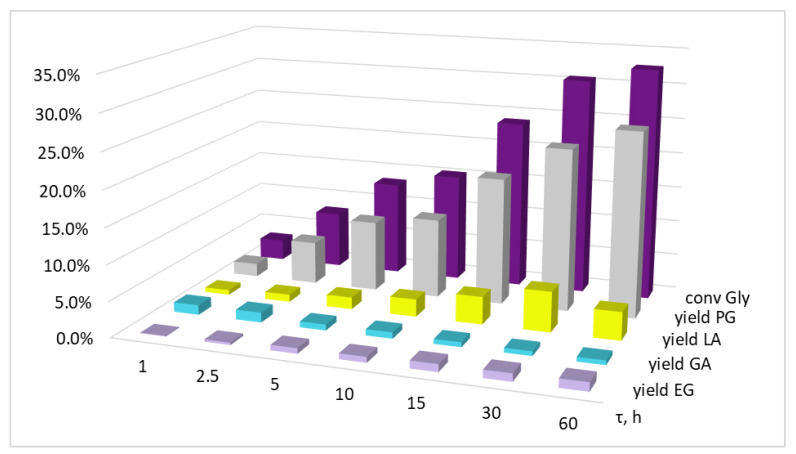
The influence of the reaction time on glycerol conversion and the yields of the products. Conditions: T = 220 °C, p(H_2_) = 3.0 MPa, KOH/Cu = 2.3 mol, Gly/Cu = 50 mol, Gly/H_2_O = 4.1 vol, precursor salt = Cu(OAc)_2_.

The dependence of glycerol conversion and product yields on the reaction time was studied for two different catalytic systems differing in their KOH/Cu molar ratio (2.3 and 5.8) (Figure 5 and Figure 6). In both cases, the continuous growth of X_Gly_, Y_PG_ and Y_LA_ was observed. In general, the yield of the by-product ethylene glycol increased with an increase in the PG yield, while the GA yield tended to somewhat decrease. The latter circumstance can be explained by the fact that GA formed during the copper reduction by glycerol was further converted—presumably as a result of slow hydrogenation into glycerol.

With that, according to the data obtained, a sharp reaction deceleration is evident, and is more pronounced in the case of KOH/Cu = 5.8 mol (Figure 5). Thus, the change in conversion rate for the first hour of reaction was 12.6%, while for the next 29 h, the conversion rate changed by only 11.8%. In the case where KOH/Cu = 2.3 mol, the reaction deceleration was smoother: X_Gly_ at 1 and 30 h of the reaction was 2.9% and 30.6%; however, when the reaction continued for up to 60 h, the conversion increase was only 2.1% (Figure 6).

In principle, such a sharp reaction deceleration can be due to three main reasons. The first of them is the accumulation of an inhibitor compound in the reaction medium, which sharply slows down the target reaction. Theoretically, the reaction product can also act as an inhibitor, but the repeatedly described possibility of obtaining high yields of propylene glycol in the glycerol hydrogenolysis on copper catalysts indicates a low probability of such a scenario [7,25]. The second reason for reaction deceleration is to reduce the concentration of alkali by converting it to potassium lactate. When Y_LA_ reaches approximately 8%, the reaction medium becomes acidic. As is shown in Figure 2, when pH < 7, the conversion of glycerol and the yields of the main products—propylene glycol and lactic acid—are sharply reduced. The third potential cause may be the catalytic phase degradation. It is generally assumed that dispersed metal particles are well stabilized by a polyol medium; however, this does not guarantee a complete halt in agglomeration and recrystallization processes. It should be noted that the catalyst formation from the glycerol solution of the precursor salt coincides in time with the start of the catalytic reaction, and then these two processes (the catalyst evolution and the catalytic reaction) proceed in parallel. Since the main regularities of the catalytic reaction have already been described in this study, it is necessary to continue to characterize the catalyst formed in the reactor while making an attempt to describe its evolution.

### 2.2. In Situ-Generated Catalyst Characterization

The first questions to be answered in order to characterize the catalyst are: what is the precursor conversion degree to form a solid phase and what is the residual concentration of dissolved copper in the reaction mixture after the experiment? To answer these questions, the obtained reaction mixtures were subjected to thorough centrifugation; the copper concentration in the supernatants was determined by the XRF method (Table 2). The estimated copper concentration in the initial solution was 2.93 wt %. In the reaction mixture supernatant obtained with a reaction time of 1 h, the total residual copper content was 7 × 10^−4^ wt %. The reaction mixture obtained at τ = 5 h was characterized by ωCu = 1 × 10^−4^ wt %; there was slightly more residual copper during the solid phase formation in the absence of alkali (11 × 10^−4^ wt %). Thus, we can speak about the complete precursor salt conversion into a heterogeneous phase under the selected reaction conditions.

**Figure 7:**
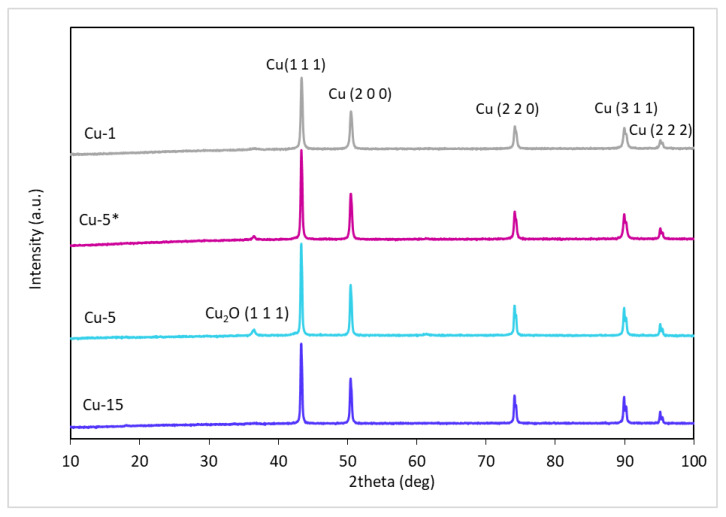
The XRD patterns of copper catalysts (Cu-1, Cu-5 *, Cu-5, Cu-15) generated in situ in the reaction medium during hydrogenolysis of glycerol.

According to the X-ray diffraction pattern obtained, the phase composition of the formed catalyst was a crystalline metallic copper Cu^0^ with a typical face-centered cubic crystal lattice (Figure 7). The observed diffraction maximum at 2θ = 43° corresponded to a set of Cu (1 1 1) copper planes (PDF 04-0836). Traces of phases other than metallic copper were represented by weak peaks of copper hemioxide (PDF 65-3288); the most intense peak was observed at 2θ = 37°, corresponding to Cu_2_O (1 1 1), the presence of which may be due to partial oxidation of the surface of solid particles during their preparation for analysis. The phase composition was the same for samples obtained in the absence of alkali and at KOH/Cu = 5.8; no differences in the phase composition were found between samples obtained after 1, 5 and 15 h of the reaction.

**Figure 8:**
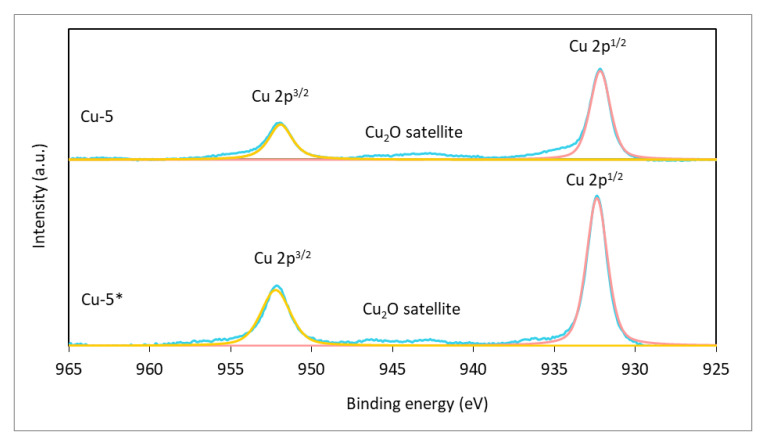
The XPS patterns of copper catalysts (Cu-5 *, Cu-5) generated in situ in the reaction medium during hydrogenolysis of glycerol. Blue color: the measured XPS spectrum; pink color: Cu 2p^1/2^ region; yellow color: Cu 2p^3/2^ region.

In view of there being no significant differences between the XRD patterns of Cu samples, XPS patterns were obtained solely for Cu-5 and Cu-5 * with a reaction of time 5 h (Figure 8). A study of the near-surface layer of in situ-formed, dispersed catalyst using the XPS method revealed the presence of Cu^0^, as evidenced by the 2p^1/2^–2p^3/2^ spin–orbit doublet, and did not identify the CuO oxide phase. As a result of the obtained XPS spectrum deconvolution, Cu^0^ peaks were identified with Cu 2p^3/2^ binding energies and Cu 2p^1/2^ equal to 932.1 eV and 951.7 eV, which is consistent with the literature data [38]. Due to the proximity of binding energies at the 2p level of Cu_2_O and Cu, which leads to the superposition of peaks, it is only possible to determine the content of copper hemioxide from the Cu_2_O satellite peak (E_bin_ = 945 eV). Thus, the surface layer of Cu-5 and Cu-5 * samples was represented by pure Cu^0^ copper with an insignificant content of copper hemioxide in the Cu-5 sample, the formation of which could be caused by sample preparation for analysis.

**Figure 9:**
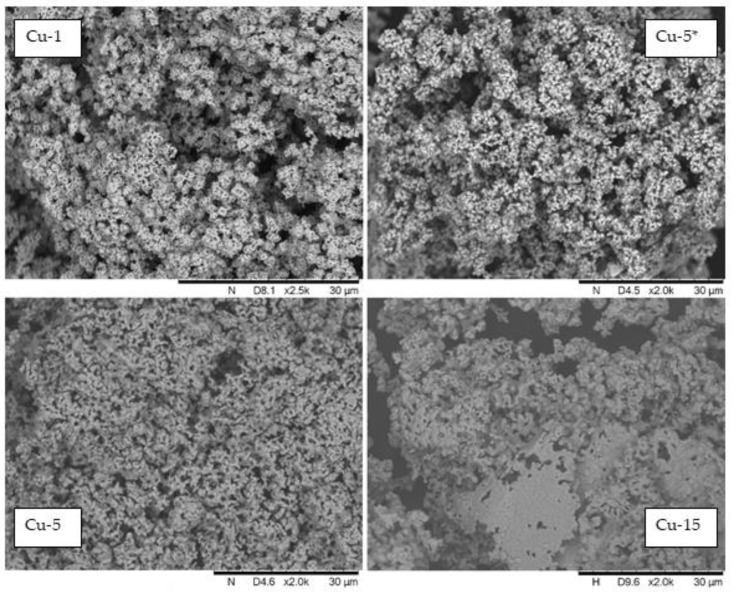
The SEM microphotographs of the copper catalysts (Cu-1, Cu-5 *, Cu-5, Cu-15) generated in situ in the reaction medium during hydrogenolysis of glycerol.

Figure 9 shows the SEM images of the catalysts Cu-1, Cu-5 and Cu-15 synthesized in situ for 1, 5 and 15 h, respectively, with KOH/Cu = 5.8 mol and Cu-5 * generated for 5 h. The morphology of all of the samples presents aggregates of various-shaped particles. According to SEM images of the catalysts Cu-1, Cu-5 and Cu-15, the particle shape changed depending on the sample formation time in situ. The average size of aggregates was 50–750 µm and the size of smaller particles was less than 5 µm (Figure S3A,B). The SEM microphotographs allowed us to make a suggestion about the evolution of the catalyst and the changes in the morphology of both the aggregates as a whole and the morphology of individual particles that made up the aggregates. For a reaction time of more than 1 h, gradual recrystallization of the copper phase resulted in the formation of agglomerates that were 50–750 µm in size, and the morphology of individual particles changed from framework- to monolith-like, probably as a result of the internal space of the framework being filled with new copper atoms (sample Cu-5, Figure 9 and Figure S3C). Some areas of the catalyst sample Cu-15 became a monolithic surface, increasing the reaction time to 15 h (sample Cu-15, Figure 9). The fact that agglomeration of the catalysts took place during this time is also confirmed by the values of the total specific surface area obtained using the BET method. For 1 h of sample Cu-1, the value was 2.75 m^2^/g; for a 15 h catalyst Cu-15, the value was more than two-times lower—0.74 m^2^/g. The process of sintering continued, and after 60 h, the catalyst had the form of a monolithic sphere (Figure S4).

**Figure 10:**
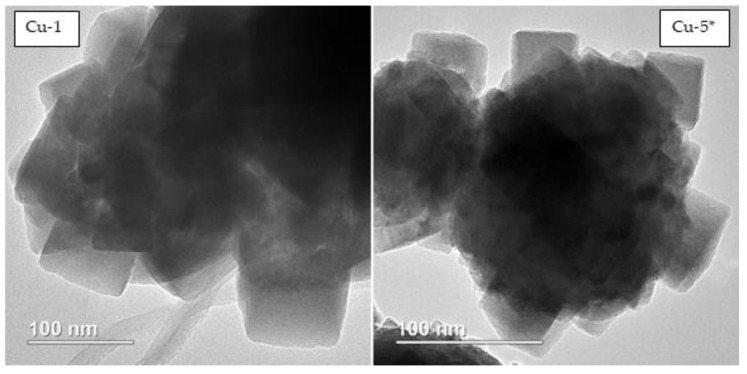
The HRTEM microphotographs of the copper catalysts (Cu-1, Cu-5 *) generated in situ in the reaction medium during hydrogenolysis of glycerol.

The absence of an alkali addition to the reaction system should be noted, and, accordingly, forming the catalyst does not lead to a significant change in morphology (sample Cu-5 *, Figure 9). Hence, sintering of the catalyst occurs during the course of the reaction time, and the active surface of the catalyst and its catalytic activity decreases, leading to a decrease in the rate of the glycerol hydrogenolysis reaction (Figure 5 and Figure 6). Catalyst microstructure analysis using the TEM method (Figure 10) indicates the formation of cubic-shaped particle clusters. Taking into account the particle size, distribution from the HRTEM method is not evident, and one can estimate that agglomerates consist of cubic particles of approximately 50–70 nm in size.

The conclusions drawn regarding the catalyst evolution from the SEM results were confirmed by the calculations of the average crystallite size from the XPA data (Table 3). The sizes of Cu crystallites were estimated by the width of the patterns, appropriate to the (111), (200), (220), (311), (222) planes in Cu according to the Hall–Williamson method. Thus, the catalyst obtained after 1, 5 and 15 h is characterized by an average crystallite size of 35 nm, 44 nm, and 49 nm, respectively.

### 2.3. The Discussion Regarding the Reaction Routes and the Specific Activity

Upon glycerol hydrogenolysis of in situ-generated dispersed copper particles, four products were formed. The main reaction products were PG and LA; by-products were represented by EG and glyceric acid. The data obtained as a result of catalytic experiments in combination with published data allow us to make assumptions regarding the routes of formation of glycerol conversion products.

The glycerol conversion reaction rate increased sharply during the transition from a neutral medium to an alkaline one (Figure 2). With that, the copper catalyst activity in a weak-acid medium was several times lower compared with the specific activity of the promoted Cu/Cr_2_O_3_ catalyst, which confirms the previous conclusions by Nikolaev et al. [7]. This allows us to conclude that the reaction mechanism changes during the transition to an alkaline medium. It is known that in a neutral and acidic medium, the dominant conversion mechanism involves the primary glycerol dehydration with the acetol formation, which is converted to PG during hydrogenation [6,7,22,25]. During the reaction by this mechanism, intermediate acetol is always detected among the by-products. In an alkaline medium, the mechanism of glycerol conversion includes primary dehydration dehydrogenation with the formation of glyceraldehyde, followed by its dehydration into methylglyoxal (pyruvaldehyde). The latter could be either hydrogenated into PG or transformed into LA by hydration rearrangement [22,39]. Under the conditions we have chosen in an alkaline medium, the glycerol conversion proceeds according to the second mechanism through the glyceraldehyde formation; this is confirmed both by the presence of PG and LA as the main products, and by the complete acetol absence in the reaction mixtures (Figure S2A). In addition, in the presence of a Cu/Cr_2_O_3_ catalyst, the LA yield was insignificant in a neutral medium and sharply increased with KOH addition (Table 1, entry 29), which also indicates a change in the reaction mechanism with alkali addition.

**Scheme 1:**
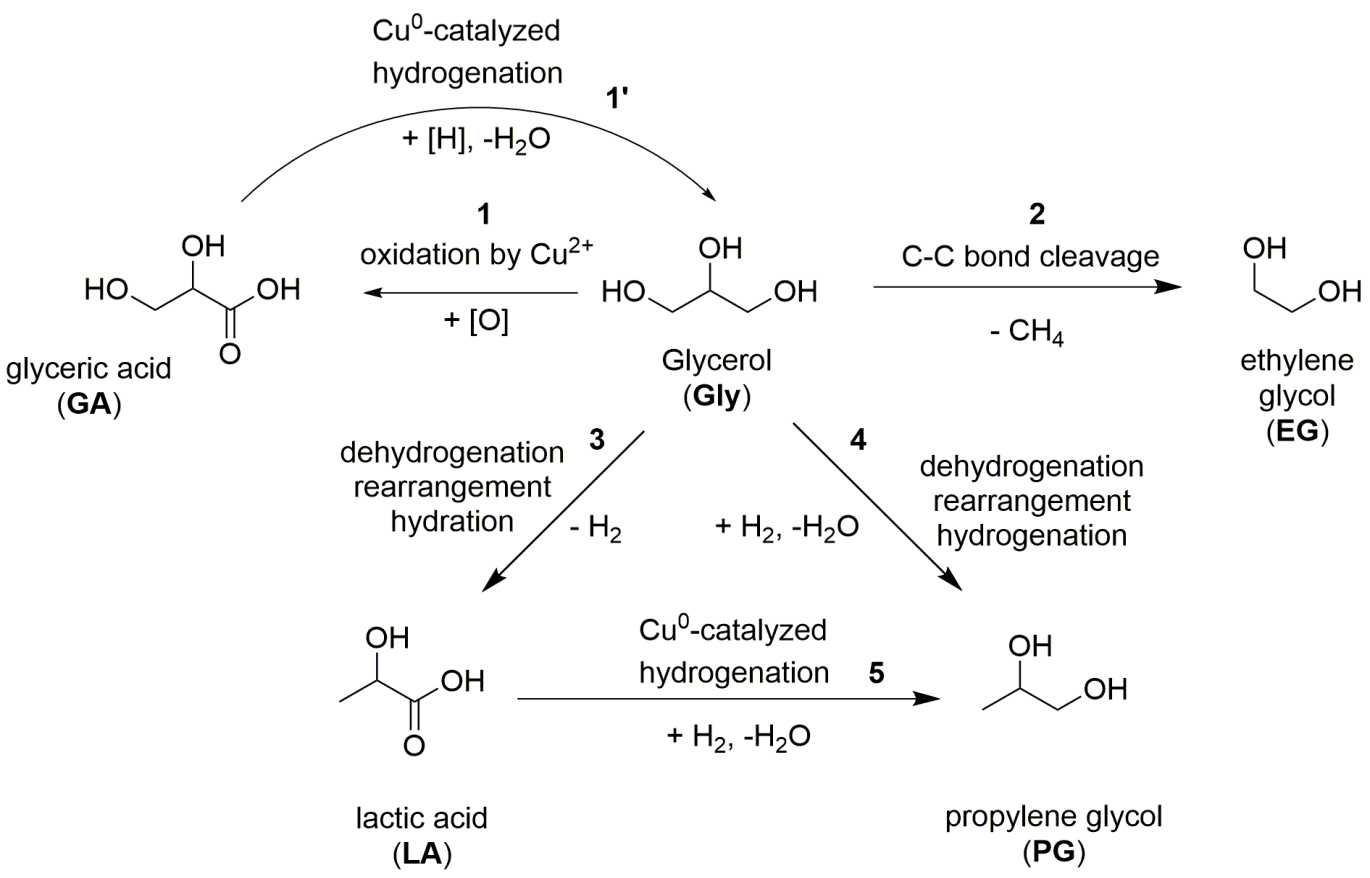
The reaction routes in the catalytic hydrogenolysis of glycerol over the in situ-generated CuDPs.

The secondary ethylene glycol formation (Scheme 1, reaction 2) during the glyceraldehyde mechanism implementation proceeded through the retro-aldol reaction of glyceraldehyde [22,39] This mechanism is characterized by a significantly higher selectivity for EG compared with the reaction occurring through acetol formation. For example, during hydrogenolysis on a Cu/Al_2_O_3_ catalyst (T = 200 °C, p(H_2_) = 5 MPa), the selectivity for ethylene glycol was 1.2% at X_Gly_ = 85%. Glycerol hydrogenolysis on Pt/C and Ru/C in the presence of CaO base (T = 200 °C, p(H_2_) = 4 MPa) proceeded with EG selectivity from 9% to 16% [22]. During glycerol hydrogenolysis in the presence of copper DPs, ethylene glycol selectivity did not depend much on pressure and the KOH/Cu molar ratio, and in all cases, was approximately 4%, practically unchanged depending on the glycerol conversion.

Glyceric acid, detected among the reaction products in all mixtures obtained by catalysis by copper DPs, is a glycerol oxidation product (Scheme 1, reaction 1). The reaction medium was reducing; therefore, the only oxidizing agent in it could be a copper salt. Since there were no other oxidizing agents in the reaction medium, GA was formed during the copper reduction from the precursor salt at the initial stage of the reaction. As the reaction continued (Figure 4 and Figure 5), the GA yield decreased slowly, which may indicate its hydrogenation with the glycerol formation in the presence of CuDPs (Scheme 1, reaction 1). Although glyceric acid could theoretically be generated by the Cannizzaro reaction of the intermediate glyceraldehyde, the main source of glyceric acid formation was considered the oxidation of glycerol by the Cu^2+^.

Since glyceric acid can be hydrogenated under reaction conditions to form glycerol, it is possible to assume that the lactic acid hydrogenation with the PG formation can proceed in a similar way (Scheme 1, reaction 5). A decrease in the CuDPs activity did not allow high glycerol conversions to be achieved where, theoretically, a secondary reaction of LA hydrogenation into PG could be observed. Nevertheless, indirect confirmation of the occurrence of this reaction was provided by data on the effect of hydrogen pressure on the product yields (Figure 2); with an increase in p(H_2_) from 1.0 to 4.0 MPa, LA selectivity decreased from 45.8 to 28.5 mol % (T = 220 °C, Gly/Cu = 50 mol, τ = 5.0 h, precursor salt= Cu(OAc)_2_).

The issue of comparing the activity of CuDPs and the industrial Cu/Cr_2_O_3_ catalyst deserves special consideration. According to Dasari et al. [17], the specific productivity for unpromoted copper heterogeneous catalysts for copper hydrogenolysis (for example, skeleton copper) is 1.5–2 times lower than for the copper chromite catalyst.

It can be assumed that the degradation rate of the catalyst, during which a decrease in dispersion and activity loss occur, is directly proportional to the concentration of copper particles in the reaction medium and temperature; as a result of which, higher relative activities of CuDPs should be observed at lower temperatures. The data obtained during the reaction at T = 200 °C show that the specific activity of the CuDPs in glycerol conversion (SP_Gly_ = 17.3–23.6 mmol g^−1^ h^−1^) was significantly higher than the activity of the Cu/Cr_2_O_3_ catalyst (SP_Gly_ = 6.5 mmol g^−1^ h^−1^) by 2.7–3.6 times (Table 4). Since 40–55% of glycerol was converted into lactic acid in the presence of the catalytic system CuDPs + KOH, the activity of dispersed particles in propylene glycol was significantly lower. For instance, for the Cu(OAc)_2_-derived DPs, the SP_PG_ amounted to 9.4 mmol g^−1^ h^−1^, which is still 1.8 times greater than the activity of the industrial catalyst (SP_PG_ = 5.3 mmol g^−1^ h^−1^). With that, it is necessary to understand that in this case, KOH acts as a co-catalyst, the mass of which is ignored when calculating SP. The activity of the Cu/Cr_2_O_3_ catalyst was measured in a catalytic run without KOH addition.

The activities of CuDPs and the Cu/Cr_2_O_3_ catalyst were also compared in experiments without free KOH at T = 220 °C (Table 5). The activity of the copper chromite catalyst almost tripled when the temperature increased from 200 to 220 °C (SP_Gly_ = 6.5 and 16.7 mmol g^−1^ h^−1^, respectively), while the activity of CuDPs was 26–36% higher than this value. With that, both catalysts showed similar values of propylene glycol activity SP_PG_.

The addition of alkali (5.8 eq) led to an increase in the activity of the Cu/Cr_2_O_3_ catalyst: the SP_Gly_/SP_PG_ increased from 16.7/15.2 to 30.4/20.8 mmol g_catalyst_^−1^ h^−1^, respectively (Table 6). The SP values for the CuDPs derived both from CuSO_4_ and Cu(OAc)_2_ were somewhat lower. However, as was shown earlier (Figure 4), for the catalytic system [Cu(OAc)_2_ + 5.8 KOH], a sharp decrease in activity was observed during the reaction, which is associated with the catalyst degradation. Obtained SP values for the reaction time of 1 and 2.5 h confirm this hypothesis; thus, according to the results after 1 h of the reaction, the SP_Gly_ and SP_PG_ for CuDPs were 3.26 and 2.61 times higher than those for the Cu/Cr_2_O_3_, respectively. It should be pointed out that the SP values are close for different copper salts (acetate, sulfate, chloride) under the same reaction conditions; thus, from the point of view of catalytic activity, the type of salt to be used in the hydrogenolysis of glycerol is not significant. However, it is possible that the choice of anion can have a long-term impact on reactor performance.

It can be concluded that, for the successful application of the in situ-generated CuDPs, it is necessary to solve the problem of catalyst degradation. Obviously, the polyol medium cannot restrain processes of copper coagulation and recrystallization under the selected conditions, which are associated with both a high reaction temperature and a relatively high concentration of dispersed particles. Possible solutions may include the use of active particle dispersion stabilizers or the formation of non-monometallic particles stabilized by the second component (e.g., metal oxide).

## 3. Materials and Methods

### 3.1. Materials

Copper (II) acetate monohydrate (98%, Komponent-Reaktiv, Moscow, Russia), Copper (II) sulfate pentahydrate (98%, Ruskhim, Moscow, Russia) and Copper (II) chloride dihydrate (98%, Komponent-Reaktiv, Moscow, Russia) were used as precursors for ultrafine copper particle formation without any further purification. Potassium hydroxide (98%, Chimmed, Moscow, Russia), glycerol (≥99.3%, Komponent-Reaktiv, Moscow, Russia) and distilled water were used as reaction feeds. In addition, 1,2-Butanediol (98%, abcr, Karlsruhe, Germany) was used as the inner standard, and 1,2-propanediol (>99%, Carl Roth, Karlsruhe, Germany), ethylene glycol (≥99.5%, Komponent-Reaktiv, Moscow, Russia), DL-lactic acid in aqueous solution (80%, Komponent-Reaktiv, Moscow, Russia) and hydroxyacetol (technical, Acros organics, Austria) were used for the GC quantification method. Trimethylsilylating reagent TMS-HT (hexamethyldisilazane + trimethylchlorosilane in anhydrous pyridine; abcr, Karlsruhe, Germany) was used for derivatization. For all hydrogenation processes, hydrogen gas (grade A in accordance with GOST 3022-80, MGPZ, Moscow, Russia) was used. For comparison purposes, a commercial copper chromite catalyst, VNH-103 (Vniineftekhim-103), that contained approximately 56% copper was used. The catalyst was reduced in hydrogen flow (10 vol % H_2_/Ar, 6 h at 300 °C) before use.

### 3.2. Catalytic Test

The liquid phase catalytic tests were performed in a stainless-steel batch reactor (50 cm^3^ internal volume) equipped with a polytetrafluoroethylene (PTFE) liner, pressure gauge, a thermocouple and a magnetic stirrer. The reactor was loaded with aliquots of glycerol and water, and either a charge of copper chromite catalyst or a precursor composition consisting of a copper salt and KOH in specified quantities. After the loading of the reactor with the feed, the precursor salt/solid catalyst, the alkali and the stirrer, the reactor was purged twice with 3 MPa of hydrogen and then filled with an operating pressure of hydrogen.

The closed autoclave was placed in an electric oven and stirring was turned on; this moment was taken as the beginning of the reaction. The set temperature (200 or 220 °C) inside the reactor was reached, on average, within 30–40 min. The ratios of the glycerol volume over the water volume (0.4–4.1 vol), mass of potassium hydroxide, reaction time (1–60 h) and hydrogen pressure (1–4 MPa) were varied at the constant total volume of the reaction mixture and at the molar ratio glycerol over copper, mainly 50 or 100 mol. The complete datasets can be found in the Supplementary Materials (Tables S1–S3).

The stirrer was set to 1000 rpm (the absence of diffusion limitations at this stirring speed was proven by separate tests). At the end of the test, the reactor was taken out of the electric furnace and promptly chilled with cold water. The pressure was then gently released, and the catalyst was separated from the reaction mixture by centrifugation, washed twice with distilled water and subsequently twice with isopropyl alcohol, dried under room temperature in dry Ar flow and stored under an inert Ar atmosphere prior to the characterization analyses.

In order to check the quantity of the reaction gaseous products, the reactors were weighed twice: before the catalytic test and after the end of the test, as the reactors were cooled and the pressure was released. The carbon balance in all of the experiments was greater than 100 ± 5%. The gaseous product yield was found to be negligible and therefore did was not included in further calculations.

### 3.3. Analysis of Products

The GC-FID analysis of the reaction products was carried out using a Krystallux 4000M chromatograph equipped with a flame ionization detector, the capillary column Optima-1 (25 m × 0.32 mm, film thickness 0.35 μm) and helium as the carrier gas. The temperature programming mode was as follows: 70 °C—holding for 1 min; from 70 to 100 °C; with a temperature rise rate of 3 deg min^–1^; 100 °C—holding for 1 min; from 100 to 230 °C with a temperature rise rate of 30 deg min^–1^; and 230 °C—holding for 1 min. Calibration for quantitative analysis by the internal standard method was conducted using standard samples of the compounds and 1,2-butanediol as the internal standard (Figure S1).

The feedstock and product compositions were studied using the GC–MS technique on a ThermoFocus DSQ II instrument (Figure S2) with the capillary column Varian VF-5 ms (30 m × 0.25 mm; film thickness 0.25 μm) and helium as the carrier gas. The operating mode was as follows: the injector temperature was 270 °C, the starting temperature of the chromatograph oven was 40 °C, heating was at a rate of 15 °C/min to 300 °C and isothermal holding was for 10 min. The mass spectrometer operating mode was as follows: ionization energy was 70 eV, source temperature was 230 °C and scanning was in the range of 10–800 Da at a speed of 2 scans/s, with unit resolution over the entire mass range. For the GC–MS analysis, 10 μL of the initial mixture was diluted with 1 mL of methylene chloride (analytical grade, Himmed). The GC-MS analysis did not reveal the formation of 1,3-propanediol.

Glycerol conversion (X) and product yields (Y) were calculated using the following equations:

$$X\left(\%\right)=\frac{\mathrm{mole}\;\mathrm{of}\;\mathrm{consumed}\;\mathrm{substrate}\;}{\mathrm{initial}\;\mathrm{mole}\;\mathrm{of}\;\mathrm{substrate}}\cdot 100\%$$

$$Y\left(\%\right)=\frac{\mathrm{mole}\;\mathrm{of}\;\mathrm{formed}\;\mathrm{products}\;}{\mathrm{initial}\;\mathrm{mole}\;\mathrm{of}\;\mathrm{substrate}}\cdot 100\%$$

### 3.4. Silylation Protocol

To obtain more volatile derivatives, the reaction mixture with 1,2-butanediol as an internal standard was derivatized with the commercially available trimethylsilylating reagent TMS-HT and analyzed by GC-FID on a non-polar column. Before GC analysis, all carboxyl-, hydroxyl groups of the obtained products during glycerol hydrogenolysis, as well as anions of lactate and glycerate, were derivatized into TMS esters. For derivatization, 5 μL of the initial mixture was mixed with 350 μL of the derivatizing agent.

### 3.5. Characterization of Copper Catalysts

To identify the phase composition of catalysts synthesized, X-ray diffraction (XRD) was used. The XRD spectra were obtained with a Rigaku D/Max-RC diffractometer using Cu Kα radiation. The elemental composition of the catalyst surface was studied by XPS with a PHI 5500 ESCA X-ray photoelectron spectrometer from Physical Electronics. Nonmonochromatic AlK_a_ radiation (hn = 1486.6 eV) with a power of 300 W was used for the excitation of photoemission. Powders were pressed into an indium plate. The diameter of an analysis zone was 1.1 mm. The photoelectron peaks were calibrated based on the C^1s^ line of carbon with a binding energy of 284.9 eV. The deconvolution of spectra was performed by a nonlinear least squares method with the use of Gaussian and Lorentzian functions.

The structure and morphology of the catalyst samples were studied using high-resolution transmission electron microscopy (HRTEM) with a JEM 2100 electron microscope from JEOL at an accelerating voltage of 200 kV.

Electron microscopic images of the samples were obtained on a Hitachi TM3030 scanning electron microscope (SEM).

The surface area measurements of the samples were carried out using a Belsorp mini X device from MICROTRAC MRB. To perform the analysis, a weighed sample of ~0.2 g was placed in a preliminarily weighed glass cuvette. The stage of preliminary preparation of the samples included thermal vacuum at temperature 200 °C under pressure at 10 Pa for 8 h. The total specific surface of the samples was determined in accordance with the BET method in the range of relative pressures p/p_0_ = 0.05–0.35.

To estimate the reduction completeness from the copper precursor after the reaction, the copper content in the supernatant was measured by X-ray fluorescence (XRF, Thermo ARL 4460).

In situ-generated catalysts Cu-1, Cu-5 and Cu-15 from the Cu(OAc)_2_ precursor salt were generated under p(H_2_) = 3 MPa at 220 °C, KOH/Cu = 5.8 mol and Gly/H_2_O = 4.1 vol and reaction times of 1, 5, and 15 h, respectively, and catalyst Cu-5 * was generated under the same conditions as catalyst Cu-5, except the addition of alkali. Cu-1, Cu-5, Cu-5 * and Cu-15 were characterized by XRD, HRTEM, and SEM. 

In order to compare the rates of hydrogenolysis of glycerol between in situ-generated catalysts in a water–glycerol media and a commercially available Cu-Cr_2_O_3_ catalyst, the values of the specific productivity (SP) were calculated using the following Equation:

$$\mathrm{SP}=\frac{n_{\mathrm{PG}}}{m_{\mathrm{cat}\cdot h}},\;\mathrm{mmol}\;g^{-1}\;h^{-1}\;$$

(mmol of PG formed per gram of the catalyst per hour).

## 4. Conclusions

In this paper, the reaction of glycerol hydrogenolysis in the presence of in situ-generated Cu dispersed particles (CuDPs) was studied. It was found that during the decomposition of precursor salts (CuSO_4_, CuCl_2_, Cu(OAc)_2_) in glycerol under the conditions of a hydrogenolysis reaction (T = 200–220 °C, p(H_2_) = 1.0–4.0 MPa), copper dispersed particles (CuDPs) were formed and catalyzed the hydrogenolysis of glycerol to give propylene glycol and lactic acid. By-products of the conversion were ethylene glycol and glyceric acid, and the latter was formed during the copper reduction from precursor salts and was hydrogenated into glycerol as the reaction continued. The effect of reaction conditions was described (T, p(H_2_), KOH/Gly and Gly/H_2_O ratios) for glycerol conversion and product yields. It was shown that an increase in the pressure and glycerol concentration increased the PG yield with practically no effect on the LA yield. The CuDPs activity at KOH/Cu < 2 ratios was very low (Y_PG_ = 0.8–1.1%, Y_LA_ = 0.6–1.1%, T = 220 °C), increasing abruptly during the transition to a slightly alkaline medium (KOH/Cu = 2.3, Y_PG_ = 10.0%). Consequently, the lactic acid yield remained relatively low (Y_LA_ = 0.6–1.1%). A further increase in the alkali amount to KOH/Cu = 5.8 mol was accompanied by a significant increase in the selectivity for lactic acid (Y_LA_ = 6.8%) without an increase in PG yield (Y_PG_ = 8.5%). The alkalinity of the reaction media therefore allows the reaction mechanism to be tuned.

Samples of CuDPs were further characterized by XRD, XPS, SEM and HRTEM. The catalysts were found to consist of pure metallic copper with a tiny admixture of Cu_2_O. According to the results of the analysis of morphology by electron microscopy, the catalyst consisted of agglomerates of an irregular shape with a mean size of 50–750 μm. Agglomerates consisted of cubic units measuring approximately 50–70 nm in size. During the reaction, the catalyst evolved, consisting of the sintering and recrystallization of copper particles, which resulted in a decrease in dispersion and activity loss. Significant activity loss had already been observed by the 5th hour of the existence of CuDPs.

Based on the results obtained, the formation methods of the main products (PG, LA) and by-products (EG, GA) were discussed. Under the selected conditions, the main reaction mechanism is presumably a glyceraldehyde-based mechanism, allowing both PG and LA to be formed. The activity of the obtained samples of CuDPs was compared with the activity of an industrial Cu/Cr_2_O_3_ catalyst. It was demonstrated that both in the presence of alkali and in a neutral medium, the specific activity of dispersed particles was higher or equal to the activity of an industrial catalyst. The highest activity for the [CuPDs + KOH] catalytic system was recorded at reaction time τ = 1 h (SP_Gly_ = 99.2, SP_PG_ = 54.3 mmol g^−1^ h^−1^); under the same conditions, at τ = 5 h, the activity of the Cu/Cr_2_O_3_ catalyst was substantially lower (SP_Gly_ = 30.4, SP_PG_ = 20.8 mmol g^−1^ h^−1^).

## Figures and Tables

**Table 1:** Hydrogenolysis of glycerol in the presence of copper catalysts. Conditions: p(H_2_) = 3 MPa, KOH/Cu = 5.8 mol, Gly/H_2_O = 4.1 vol.

Entry	Precursor Salt	T, °C	Gly/Cu, mol	τ, h	X_Gly_, %	Y_EG_, %	Y_PG_, %	Y_LA_, %	Y_GA_, %
1	-	220	-	5	2.2	0.1	0.2	2.0	-
2	CuSO_4_·5H_2_O	200	50	5	15.0	0.4	7.0	7.0	0.6
3				10	15.2	0.5	8.9	5.0	0.8
4			100	5	7.0	0.2	4.0	2.5	0.3
5				10	8.1	0.4	4.7	2.9	0.1
6		220	50	5	16.6	0.8	10.4	5.1	0.3
7				10	18.4	0.9	10.7	6.8	-
8			100	5	8.5	0.4	4.7	3.3	0.1
9				10	8.5	0.3	4.8	3.4	-
10	Cu(OAc)_2_·H_2_O	200	50	5	14.8	0.3	8.9	3.7	1.9
11				10	15.5	0.6	8.0	5.7	1.2
12			100	5	7.0	0.1	3.6	2.6	0.7
13				10	7.1	0.2	3.7	2.5	0.7
14		220	50	5	16.8	0.7	8.5	6.8	0.8
15				10	18.5	0.8	9.4	7.4	0.9
16			100	5	7.8	0.2	4.0	3.2	0.4
17				10	10.6	0.5	5.3	4.2	0.6
18	Nitrogen	200	100	5	4.6	0.1	0.7	3.1	0.7
19		220		5	4.4	0.1	0.7	2.8	0.8
20				10	4.8	0.1	0.8	3.3	0.6
21	Cu-Cr_2_O_3_^ 1^	200	50	5	7.4	0.1	6.0	1.3	-
22				10	10.6	0.1	10.1	0.4	-
23			100	5	3.6	-	3.6	-	-
24				10	9.3	0.3	8.3	0.7	-
25		220	50	5	19.0	0.2	17.3	1.5	-
26				10	26.1	0.2	25.4	0.5	-
27			100	5	11.9	0.1	8.6	3.1	-
28				10	19.4	0.1	19.0	0.4	-
29	Cu-Cr_2_O_3_ + KOH	220	50	5	34.5	1.5	23.6	9.4	-
30	CuCl_2_·2H_2_O	200	50	5	11.0	0.3	5.8	4.3	0.6
31		220	50	5	16.2	0.5	6.7	8.3	0.7

^1^ The copper chromite catalyst was used without KOH addition.

**Table 2:** The residual copper content in the supernatant solutions measured by XRF. Conditions: T = 220 °C, p(H_2_) = 3.0 MPa, Gly/Cu = 50 mol, Gly/H_2_O = 4.1 vol, precursor salt = Cu(OAc)_2_.

Reaction Conditions	ωCu^2+^ at the Beginning of the Reaction, %	∑Cu after Hydrogenolysis, 10^−4^%
τ = 5 h, Without alkali	2.93	11
τ = 5 h, nKOH/nCu = 5.8		1
τ = 1 h, nKOH/nCu = 5.8		7

**Table 3:** Phase composition and average crystallite size of in situ-generated catalysts estimated by XRD.

Catalyst	Phase Composition	Average Crystallite Size, nm
Cu-1	Cu	35
Cu-5 *		42
Cu-5		44
Cu-15		49

**Table 4:** The SP values for the various copper catalysts in the glycerol hydrogenolysis. T = 200 °C, p(H_2_) = 3.0 MPa, KOH/Cu = 5.8 mol, Gly/Cu = 50 mol, τ = 5 h.

Precursor Salt	SP_Gly_, mmol Gly g_catalyst_^−1^ h^−1^	SP_PG_, mmol PG g_catalyst_^−1^ h^−1^
CuSO_4_·5H_2_O	23.6	10.7
Cu(OAc)_2_·H_2_O	18.6	9.4
Cu-Cr_2_O_3_^ 1^	6.5	5.3
CuCl_2_	17.3	9.1

^1^ The copper chromite catalyst was used without KOH addition.

**Table 5:** The SP values for the CuDPs and the Cu/Cr_2_O_3_ catalyst in the glycerol hydrogenolysis. T = 220 °C, p(H_2_) = 3.0 MPa, KOH/Cu = 2.3 mol (for copper acetate) and 0 (for Cu/Cr_2_O_3_), Gly/Cu = 50 mol.

Precursor Salt	τ, h	SP_Gly_, mmol Gly g_catalyst_^−1^ h^−1^	SP_PG_, mmol PG g_catalyst_^−1^ h^−1^
Cu-Cr_2_O_3_	5	16.7	15.2
Cu(OAc)_2_	1	22.8	15.0
	2.5	25.2	19.2
	5	21.1	15.7

**Table 6:** The SP values for the various copper catalysts in the glycerol hydrogenolysis. T = 220 °C, p(H_2_) = 3.0 MPa, KOH/Cu = 5.8, Gly/Cu = 50 mol.

Precursor Salt	τ, h	SP_Gly_, mmol Gly g_catalyst_^−1^ h^−1^	SP_PG_, mmol PG g_catalyst_^−1^ h^−1^
CuSO_4_·5H_2_O	5	26.1	16.4
Cu-Cr_2_O_3_	5	30.4	20.8
Cu(OAc)_2_·H_2_O	1	99.2	54.3
	2.5	45.4	23.6
	5	26.6	13.2

## Data Availability

Not applicable.

## Supplementary Materials

The following supporting information can be downloaded at: https://www.mdpi.com/article/10.3390/molecules27248778/s1, Tables S1–S3: The average values of conversion of glycerol (X_Gly_), yields of products (Y) under different reaction conditions during glycerol hydrogenolysis. Figure S1: Calibration plots of response ratio (S_st_/S) versus mass ratio (m_st_/m) for internal standardization by GC-FID for A: TMS-Gly derivative; B: TMS-PG derivative; C: TMS-EG derivative; D: TMS-LA derivative. Figure S2: A: The mass spectrum of a silylated liquid sample after glycerol hydrogenolysis reaction; B: The mass spectrum of TMS-Gly; C: The mass spectrum of TMS-EG; D: The mass spectrum of TMS-PG; E: The mass spectrum of TMS-LA; F: The mass spectrum of TMS-BD; G: The mass spectrum of TMS-GA. Figure S3: The SEM microphotographs of the copper catalysts generated in situ in the reaction medium during hydrogenolysis of glycerol. A, B: Cu-5; C: Cu-15. Figure S4: The appearance of the catalyst after 60 h of glycerol hydrogenolysis. Figure S5: Cu(OAc)_2_ in a water–glycerol solution prior to adding potassium hydroxide.
