# Supplementary material for: In Situ-Generated, Dispersed Cu Catalysts for the Catalytic Hydrogenolysis of Glycerol
Source: Molecules. 2022 Dec 11;27(24):8778. doi: 10.3390/molecules27248778 (PMC9781552; doi:10.3390/molecules27248778)
Supplement: Supplementary file 1 [file molecules-27-08778-s001.zip › molecules-2065132-supplementary.pdf]

Supplementary Materials to the article

In Situ-Generated, Dispersed Cu Catalysts for the Catalytic Hydrogenolysis of Glycerol

By Iuliana Porukova, Vadim Samoilov, Dzhamulutdin Ramazanov, Mariia Kniazeva, Anton Maximov

Table S1. The average values of conversion of glycerol (**X**$_{Gly}$), yields of products (**Y**) under different reaction conditions during glycerol hydrogenolysis (*S* – standard derivation of the value on the left, N – the number of experiments). Conditions: T = 200°C, Gly/H_2O = 4.1 vol, precursor salt = $Cu(OAc)_2{\cdot}H_2O$

<table>
<tr><th>Entry</th><th>Gly/Cu, mol</th><th>KOH/Cu, mol</th><th>τ, h</th><th>p(H₂), MPa</th><th>X_{Gly},%</th><th>S_x,%</th><th>Y_{EG},%</th><th>S_y,%</th><th>Y_{PG},%</th><th>S_y,%</th><th>Y_{LA},%</th><th>S_y,%</th><th>Y_{GA},%</th><th>S_y,%</th><th>N</th></tr>
<tr><td>1</td><td rowspan="7">50</td><td>0</td><td>5</td><td>3</td><td>3.1</td><td>0.4</td><td>-</td><td>-</td><td>0.1</td><td>0.1</td><td>1.4</td><td>0.4</td><td>1.6</td><td>0.1</td><td>2</td></tr>
<tr><td>2</td><td rowspan="8">5.8</td><td rowspan="4">2.5</td><td>1</td><td>9.9</td><td>0.5</td><td>0.2</td><td>0.1</td><td>3.7</td><td>0.3</td><td>4.5</td><td>0.4</td><td>1.5</td><td>0.1</td><td>2</td></tr>
<tr><td>3</td><td>2</td><td>11.5</td><td>0.6</td><td>0.3</td><td>0.1</td><td>5.4</td><td>0.6</td><td>4.2</td><td>0.1</td><td>1.6</td><td>0.1</td><td>2</td></tr>
<tr><td>4</td><td>3</td><td>13.0</td><td>2.3</td><td>0.2</td><td>0.1</td><td>7.7</td><td>1.5</td><td>3.2</td><td>0.3</td><td>1.9</td><td>0.4</td><td>2</td></tr>
<tr><td>5</td><td>4</td><td>13.0</td><td>1.2</td><td>0.4</td><td>0.2</td><td>7.2</td><td>0.9</td><td>3.7</td><td>0.5</td><td>1.7</td><td>0.1</td><td>2</td></tr>
<tr><td>6</td><td>5</td><td rowspan="4">3</td><td>14.8</td><td>0.2</td><td>0.3</td><td>0.1</td><td>8.9</td><td>0.4</td><td>3.7</td><td>0.2</td><td>1.9</td><td>0.1</td><td>2</td></tr>
<tr><td>7</td><td>10</td><td>15.5</td><td>0.6</td><td>0.6</td><td>0.1</td><td>8.0</td><td>0.2</td><td>5.7</td><td>0.3</td><td>1.2</td><td>0.1</td><td>2</td></tr>
<tr><td>8</td><td rowspan="2">100</td><td>5</td><td>7.0</td><td>0.5</td><td>0.1</td><td>0.1</td><td>3.6</td><td>0.5</td><td>2.6</td><td>1.3</td><td>0.7</td><td>0.4</td><td>3</td></tr>
<tr><td>9</td><td>10</td><td>7.1</td><td>0.6</td><td>0.2</td><td>0.1</td><td>3.7</td><td>0.3</td><td>2.5</td><td>0.3</td><td>0.7</td><td>0.1</td><td>2</td></tr>
</table>

Table S2. The average values of conversion of glycerol (**X**$_{Gly}$), yields of products (**Y**) under different reaction conditions during glycerol hydrogenolysis (*S* – standard derivation of the value on the left, N – the number of experiments). Conditions: T = 220°C, p(H_2) = 3 MPa, precursor salt = $Cu(OAc)_2{\cdot}H_2O$

<table>
<tr><th>Entry</th><th>Gly/Cu, mol</th><th>KOH/Cu, mol</th><th>Gly/H2O, vol</th><th>τ, h</th><th>XGly,%</th><th>Sx,%</th><th>YEG,%</th><th>Sy,%</th><th>YPG,%</th><th>Sy,%</th><th>YLA,%</th><th>Sy,%</th><th>YGA,%</th><th>Sy,%</th><th>N</th></tr>
<tr><td>1</td><td rowspan="23">50</td><td>0</td><td rowspan="12">4.1</td><td rowspan="3">5</td><td>3.4</td><td>1.1</td><td>0.2</td><td>0.1</td><td>0.8</td><td>0.6</td><td>0.9</td><td>0.5</td><td>1.5</td><td>0.1</td><td>16</td></tr>
<tr><td>2</td><td>0.3</td><td>2.8</td><td>0.2</td><td>-</td><td>-</td><td>0.8</td><td>0.2</td><td>0.6</td><td>0.1</td><td>1.4</td><td>0.1</td><td>2</td></tr>
<tr><td>3</td><td>0.8</td><td>3.5</td><td>-</td><td>-</td><td>-</td><td>1.1</td><td>-</td><td>1.1</td><td>-</td><td>1.3</td><td>-</td><td>1</td></tr>
<tr><td>4</td><td rowspan="7">2.3</td><td>1</td><td>2.9</td><td>0.6</td><td>0.1</td><td>0.1</td><td>1.9</td><td>0.5</td><td>0.7</td><td>0.1</td><td>0.2</td><td>0.1</td><td>2</td></tr>
<tr><td>5</td><td>2.5</td><td>8.0</td><td>1.4</td><td>0.3</td><td>0.1</td><td>6.1</td><td>1.2</td><td>1.0</td><td>0.1</td><td>0.6</td><td>0.1</td><td>2</td></tr>
<tr><td>6</td><td>5</td><td>13.4</td><td>1.5</td><td>0.7</td><td>0.1</td><td>10.0</td><td>1.1</td><td>1.7</td><td>0.5</td><td>1.0</td><td>0.2</td><td>2</td></tr>
<tr><td>7</td><td>10</td><td>15.3</td><td>2.4</td><td>0.8</td><td>0.3</td><td>11.2</td><td>2.0</td><td>2.5</td><td>0.1</td><td>0.8</td><td>0.1</td><td>2</td></tr>
<tr><td>8</td><td>15</td><td>23.8</td><td>2.1</td><td>1.1</td><td>0.4</td><td>17.9</td><td>2.6</td><td>3.9</td><td>0.9</td><td>0.9</td><td>0.2</td><td>3</td></tr>
<tr><td>9</td><td>30</td><td>30.6</td><td>1.1</td><td>1.1</td><td>0.2</td><td>22.9</td><td>1.2</td><td>5.7</td><td>1.4</td><td>0.9</td><td>0.1</td><td>3</td></tr>
<tr><td>10</td><td>60</td><td>32.7</td><td>0.9</td><td>1.5</td><td>0.1</td><td>26.0</td><td>0.1</td><td>4.0</td><td>0.7</td><td>1.2</td><td>0.4</td><td>2</td></tr>
<tr><td>11</td><td>3.6</td><td rowspan="2">5</td><td>12.6</td><td>-</td><td>0.4</td><td>-</td><td>6.3</td><td>-</td><td>4.7</td><td>-</td><td>1.2</td><td>0.1</td><td>1</td></tr>
<tr><td>12</td><td>4.6</td><td>12.8</td><td>-</td><td>0.4</td><td>-</td><td>6.4</td><td>-</td><td>4.4</td><td>-</td><td>1.6</td><td>0.1</td><td>1</td></tr>
<tr><td>13</td><td rowspan="11">5.8</td><td>0.4</td><td rowspan="3">5</td><td>13.3</td><td>1.4</td><td>0.4</td><td>0.1</td><td>4.0</td><td>0.4</td><td>7.3</td><td>1.2</td><td>1.6</td><td>0.6</td><td>2</td></tr>
<tr><td>14</td><td>0.8</td><td>14.5</td><td>1.8</td><td>0.6</td><td>0.1</td><td>6.8</td><td>1.8</td><td>5.9</td><td>0.3</td><td>1.2</td><td>0.1</td><td>3</td></tr>
<tr><td>15</td><td>1.4</td><td>13.7</td><td>0.1</td><td>0.3</td><td>0.1</td><td>6.5</td><td>1.4</td><td>5.3</td><td>2.0</td><td>1.6</td><td>0.5</td><td>2</td></tr>
<tr><td>16</td><td>2.1</td><td>30</td><td>13.4</td><td>0.6</td><td>0.4</td><td>0.1</td><td>5.9</td><td>0.5</td><td>5.8</td><td>0.1</td><td>1.3</td><td>0.1</td><td>2</td></tr>
<tr><td>17</td><td rowspan="7">4.1</td><td>1</td><td>12.6</td><td>1.7</td><td>0.4</td><td>0.1</td><td>6.9</td><td>1.0</td><td>4.0</td><td>0.8</td><td>1.3</td><td>0.2</td><td>13</td></tr>
<tr><td>18</td><td>2.5</td><td>14.4</td><td>1.0</td><td>0.4</td><td>0.1</td><td>7.5</td><td>0.8</td><td>5.1</td><td>0.9</td><td>1.4</td><td>0.3</td><td>3</td></tr>
<tr><td>19</td><td>5</td><td>16.8</td><td>2.4</td><td>0.7</td><td>0.2</td><td>8.5</td><td>1.6</td><td>6.8</td><td>1.2</td><td>0.8</td><td>0.4</td><td>21</td></tr>
<tr><td>20</td><td>10</td><td>18.5</td><td>1.3</td><td>0.8</td><td>0.2</td><td>9.4</td><td>0.8</td><td>7.4</td><td>0.6</td><td>0.9</td><td>0.3</td><td>2</td></tr>
<tr><td>21</td><td>15</td><td>20.3</td><td>2.4</td><td>0.8</td><td>0.2</td><td>10.4</td><td>1.6</td><td>8.4</td><td>1.1</td><td>0.7</td><td>0.3</td><td>13</td></tr>
<tr><td>22</td><td>30</td><td>24.4</td><td>2.8</td><td>1.1</td><td>0.4</td><td>13.5</td><td>2.4</td><td>9.1</td><td>0.1</td><td>0.7</td><td>0.1</td><td>2</td></tr>
<tr><td>23</td><td>60</td><td>23.2</td><td>2.5</td><td>0.8</td><td>0.2</td><td>12.9</td><td>2.8</td><td>8.9</td><td>0.3</td><td>0.6</td><td>0.3</td><td>2</td></tr>
<tr><td>24</td><td rowspan="6">100</td><td>0</td><td rowspan="6">4.1</td><td rowspan="3">5</td><td>2.9</td><td>0.3</td><td>0.1</td><td>0.1</td><td>1.4</td><td>0.3</td><td>0.7</td><td>0.1</td><td>0.7</td><td>0.1</td><td>2</td></tr>
<tr><td>25</td><td>0.3</td><td>2.5</td><td>0.2</td><td>0.1</td><td>0.1</td><td>1.1</td><td>0.3</td><td>0.6</td><td>0.1</td><td>0.7</td><td>0.1</td><td>2</td></tr>
<tr><td>26</td><td rowspan="4">5.8</td><td>7.8</td><td>0.6</td><td>0.2</td><td>0.1</td><td>4.0</td><td>0.4</td><td>3.2</td><td>0.1</td><td>0.4</td><td>0.1</td><td>2</td></tr>
<tr><td>27</td><td>10</td><td>11.5</td><td>0.7</td><td>0.3</td><td>0.2</td><td>5.1</td><td>0.1</td><td>5.6</td><td>0.4</td><td>0.5</td><td>0.1</td><td>2</td></tr>
<tr><td>28</td><td>15</td><td>8.6</td><td>0.7</td><td>0.3</td><td>0.1</td><td>4.4</td><td>0.4</td><td>3.4</td><td>0.3</td><td>0.5</td><td>0.1</td><td>2</td></tr>
<tr><td>29</td><td>10</td><td>10.6</td><td>-</td><td>0.5</td><td>-</td><td>5.3</td><td>-</td><td>4.2</td><td>-</td><td>0.6</td><td>-</td><td>1</td></tr>
</table>

30	200	5.0			3.9	0.4	0.1	0.1	2.0	0.1	1.5	0.3	0.3	0.1	2
31	219	11.0			5.9	2.1	0.2	0.1	2.6	1.2	2.9	0.6	0.2	0.2	2

Table S3. The average values of conversion of glycerol (**X**$_{Gly}$), yields of products (**Y**) under different reaction conditions during glycerol hydrogenolysis (*S* – standard derivation of the value on the left, N – the number of experiments). Conditions: p(H_2) = 3 MPa, Gly/H_2O = 4.1 vol

Entry	Precursor salt or catalyst	T, °C	Gly/Cu, mol	KOH/Cu, mol	τ, h	X_{Gly},%	S_x,%	Y_{EG},%	S_y,%	Y_{PG},%	S_y,%	Y_{LA},%	S_y,%	Y_{GA},%	S_y,%	N
1	$CuSO_4·5H_2O$	200	50	5.8	5	15.0	0.1	0.4	0.1	7.0	0.2	7.0	0.2	0.6	0.1	2
2					10	15.2	1.6	0.5	0.1	8.9	1.2	5.0	0.4	0.8	0.1	3
3			100		5	7.0	-	0.2	-	4.0	-	2.5	-	0.3	-	1
4					10	8.1	-	0.4	-	4.7	-	2.9	-	0.1	-	1
5		220	50		5	16.6	1.2	0.8	0.2	10.4	1.5	5.1	1.9	0.3	0.2	2
6					10	18.4	1.8	0.9	0.2	10.7	1.2	6.8	0.7	-	-	2
7			100		5	8.5	-	0.4	-	4.7	-	3.3	-	0.1	-	1
8					10	8.5	-	0.3	-	4.8	-	3.4	-	-	-	1
9	Cu-Cr_2O_3	200	50	0	5	7.4	1.9	0.1	0.1	6.0	2.0	1.3	0.1	-	-	2
10					10	10.6	2.7	0.1	0.1	10.1	2.0	0.4	0.3	-	-	2
11			100		5	3.6	0.5	-	-	3.6	0.4	-	-	-	-	2
12					10	9.3	1.2	0.3	0.2	8.3	1.6	0.7	0.2	-	-	3
13		220	50		5	19.0	1.6	0.2	0.1	17.3	1.7	1.5	0.1	-	-	2
14					10	26.1	0.9	0.2	0.1	25.4	1.3	0.5	0.4	-	-	2
15				5.8	5	34.5	-	1.5	-	23.6	-	9.4	-	-	-	1
16			100	0	5	11.8	0.9	0.1	0.1	8.6	0.8	3.1	0.1	-	-	2
17					10	19.5	3.7	0.1	0.1	19.0	4.1	0.4	0.4	-	-	3
18		230	50		5	31.8	-	1.3	-	20.7	-	9.8	-	-	-	1
19	$CuCl_2·2H_2O$	200	50	5.8	5	11.0	3.6	0.3	0.1	5.8	3.0	4.3	0.3	0.6	0.3	2
20		220				16.2	1.0	0.5	0	6.7	0.1	8.3	1.0	0.7	0.1	2

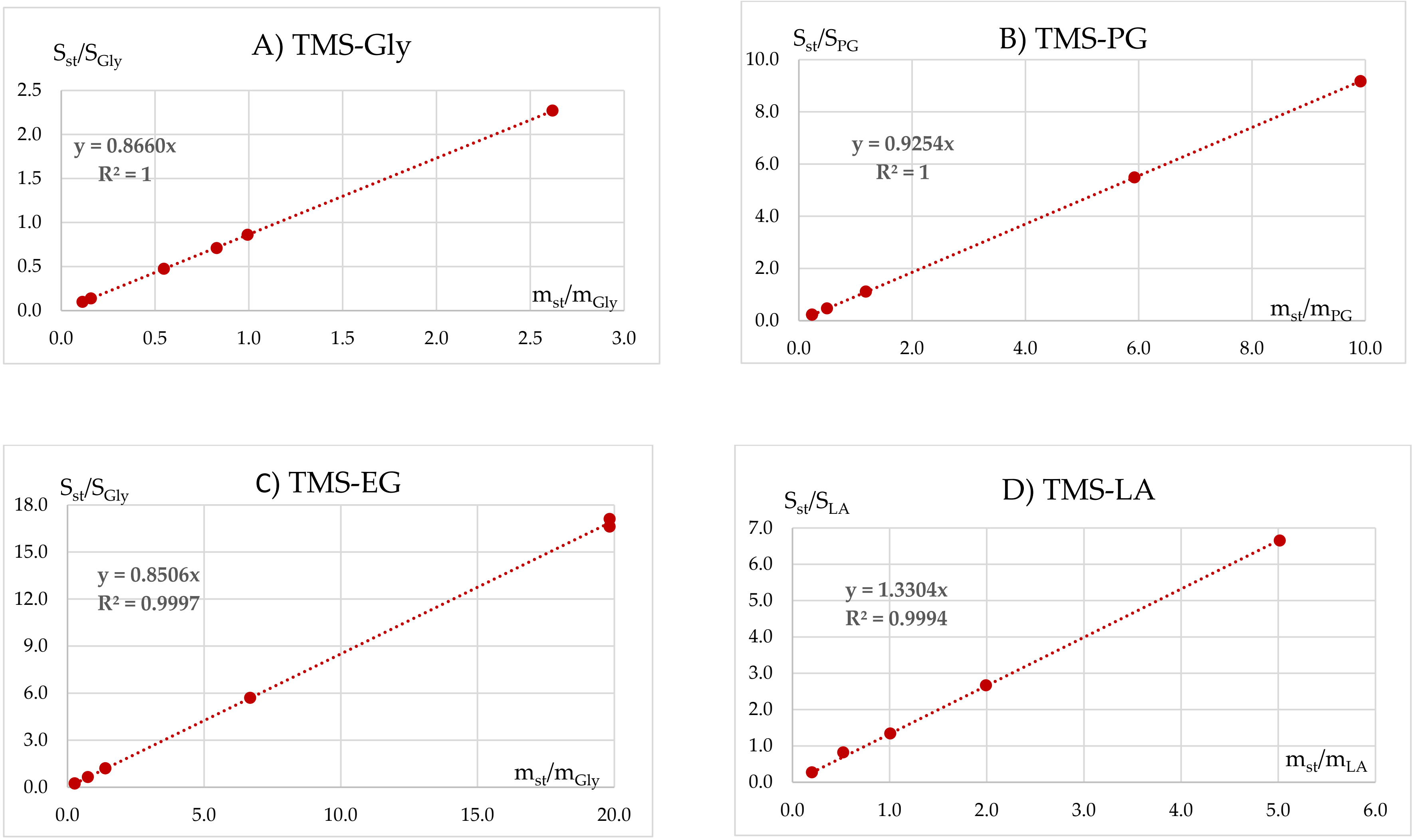

Figure S1. Calibration plots of response ratio (S_{st}/S) versus mass ratio (m_{st}/m) for internal standardization by GC-FID for A: TMS-Gly derivative; **B:** TMS-PG derivative; **C:** TMS-EG derivative; **D:** TMS-LA derivative. For GA, the same coefficient was used as for Gly.

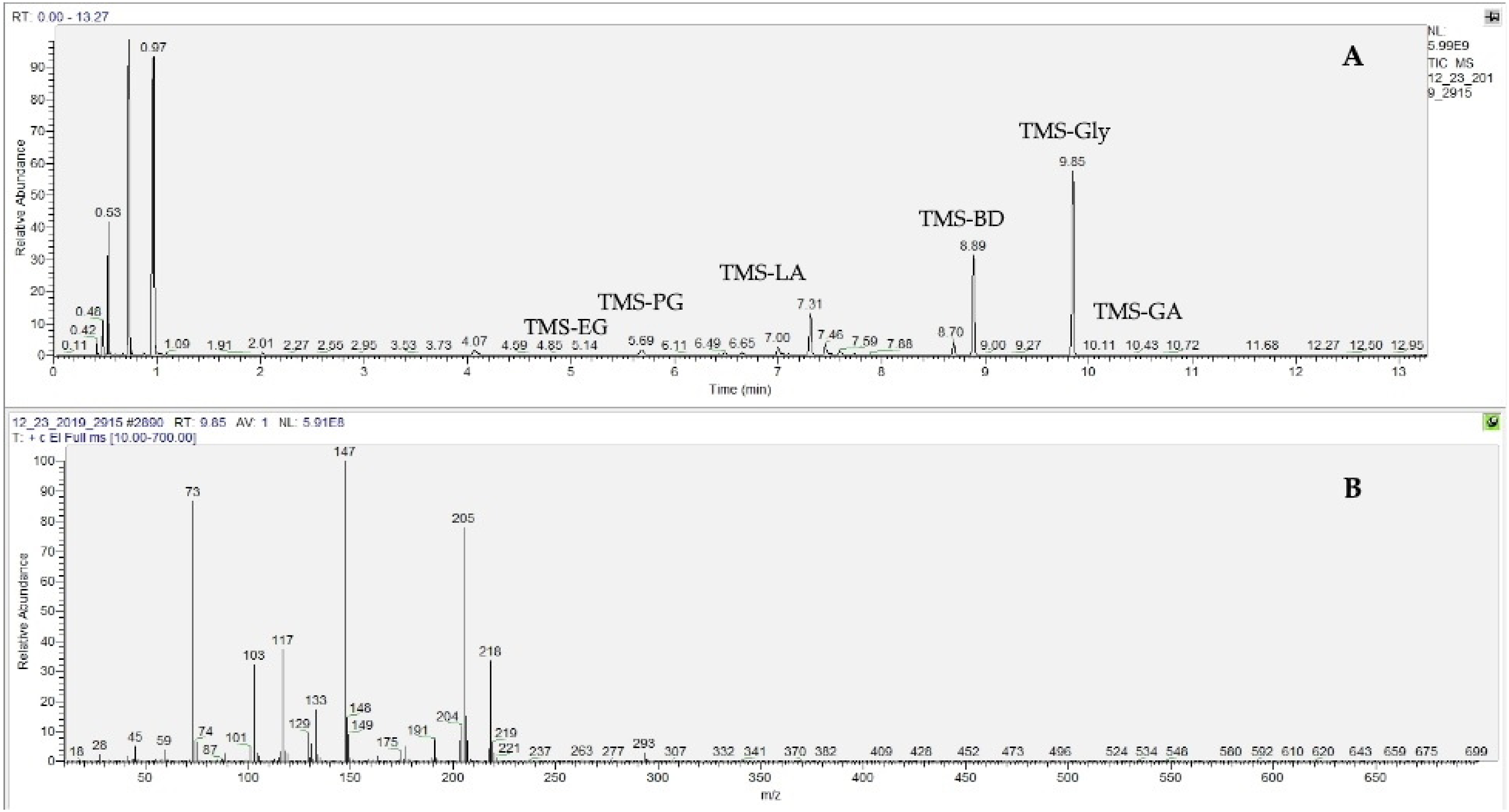
RT: 0.00 - 13.27
NL: 5.99E9 TIC MS 12_23_2019_2915
A
TMS-EG
TMS-PG
TMS-LA
TMS-BD
TMS-Gly
TMS-GA
0.11 0.42 0.46 0.53 0.97 1.09 1.91 2.01 2.27 2.55 2.95 3.53 3.73 4.07 4.59 4.85 5.14 5.69 6.11 6.49 6.65 7.00 7.31 7.46 7.59 7.88 8.70 8.89 9.00 9.27 9.85 10.11 10.43 10.72 11.68 12.27 12.50 12.95
Relative Abundance
Time (min)
12_23_2019_2915 #2890 RT: 9.85 AV: 1 NL: 5.91E8
T: + c EI Full ms [10.00-700.00]
B
18 28 45 59 73 74 87 101 103 117 129 133 147 148 149 175 191 204 205 218 219 221 237 263 277 293 307 332 341 370 382 409 428 452 473 496 524 534 546 580 592 610 620 643 659 675 699
Relative Abundance
m/z

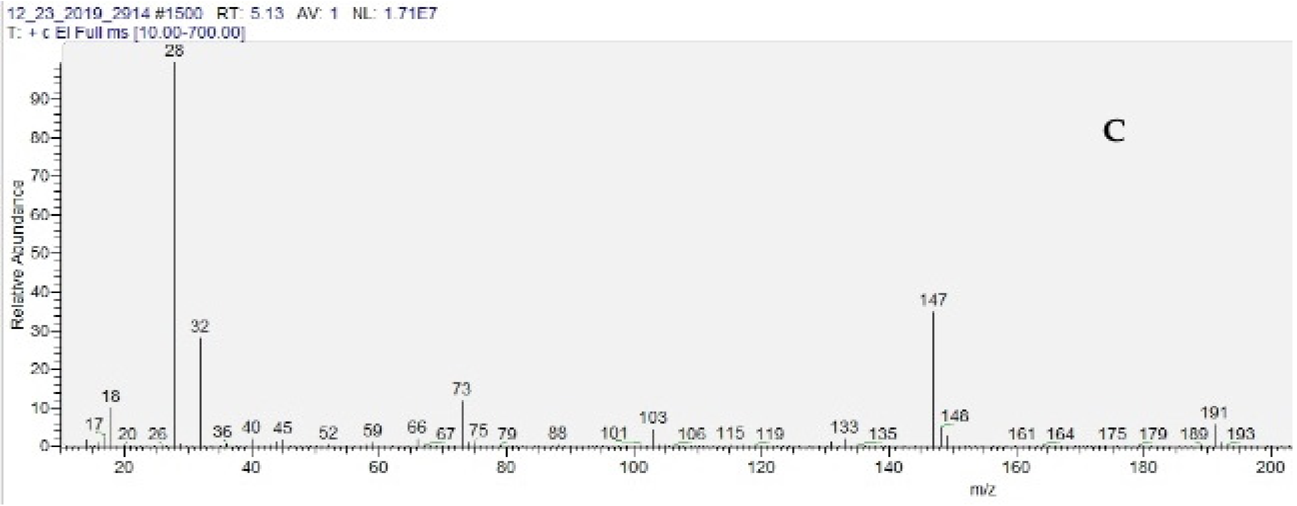
12_23_2019_2914 #1500 RT: 5.13 AV: 1 NL: 1.71E7
T: + c EI Full ms [10.00-700.00]
C
Relative Abundance
m/z

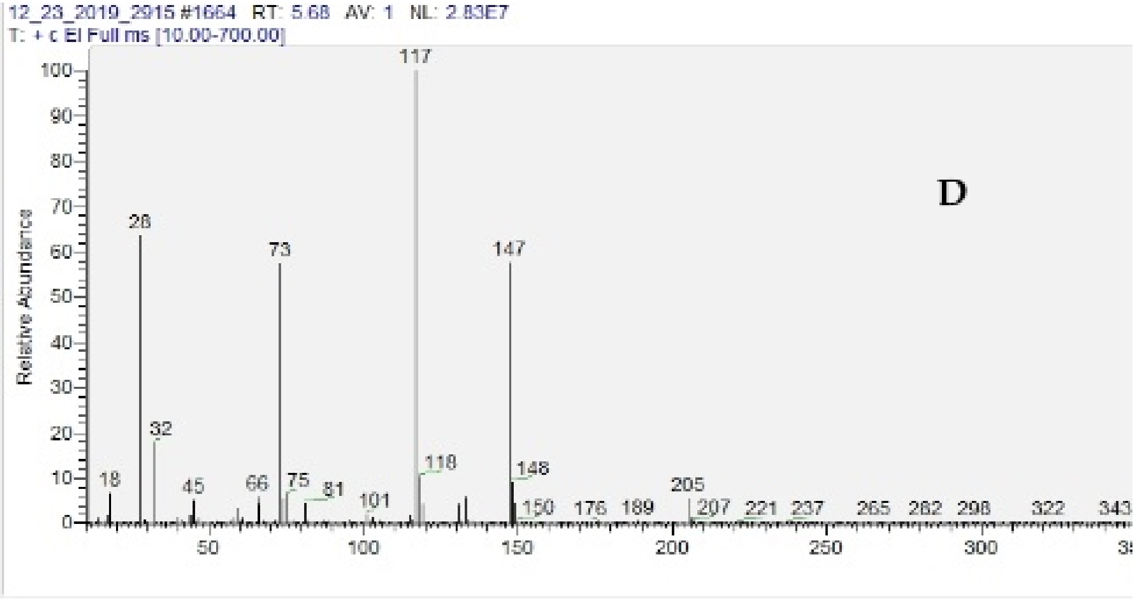
12_23_2019_2915 #1664 RT: 5.68 AV: 1 NL: 2.83E7
T: + c EI Full ms [10.00-700.00]
D
Relative Abundance

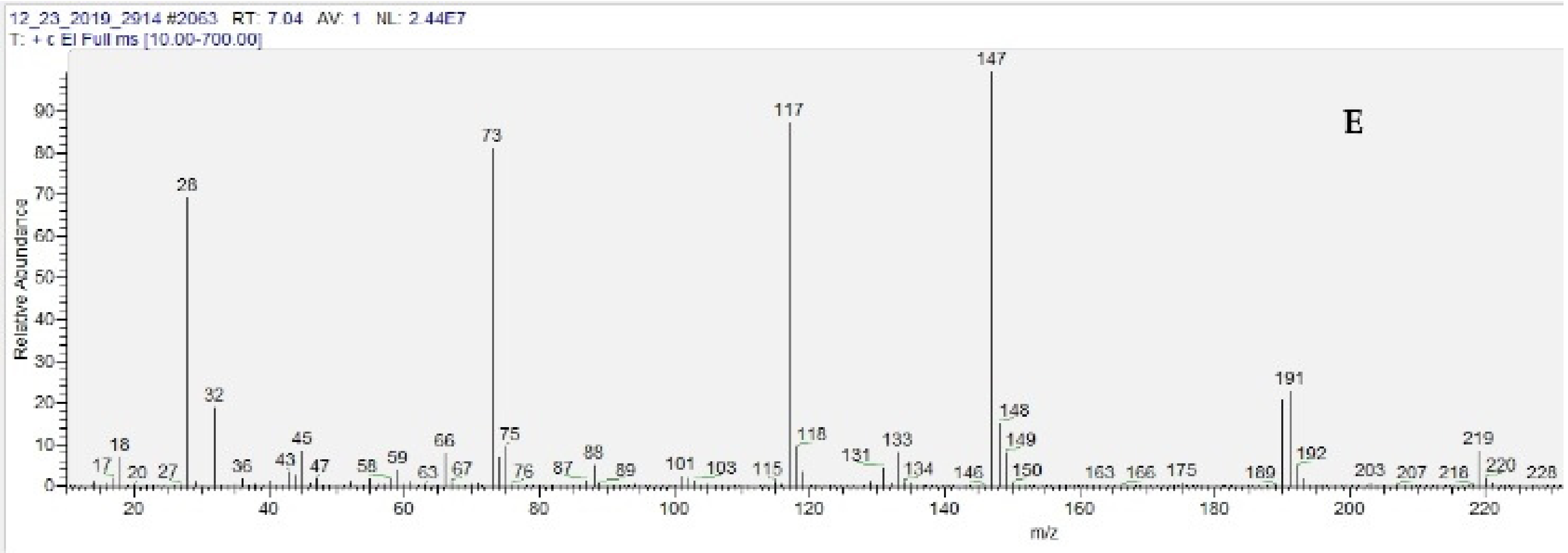
12_23_2019_2914 #2063 RT: 7.04 AV: 1 NL: 2.44E7
T: + c EI Full ms [10.00-700.00]
E
Relative Abundance
m/z

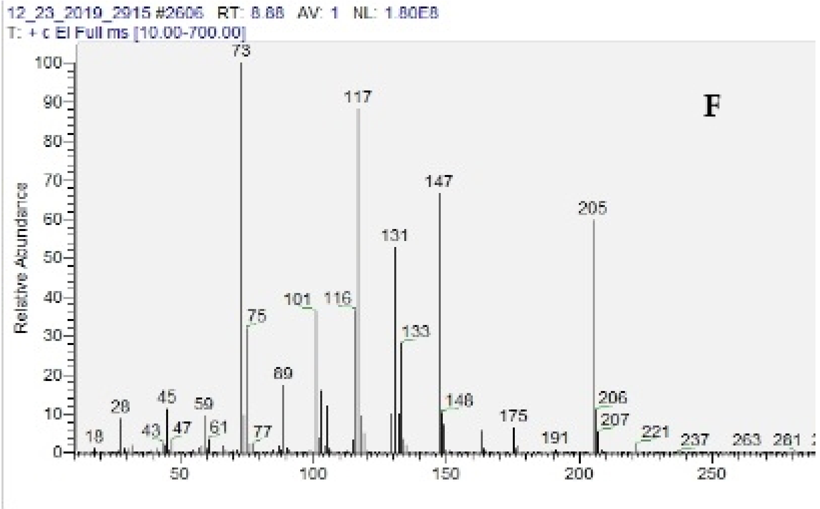
12_23_2019_2915 #2606 RT: 8.88 AV: 1 NL: 1.80E8
T: + c EI Full ms [10.00-700.00]
F
Relative Abundance

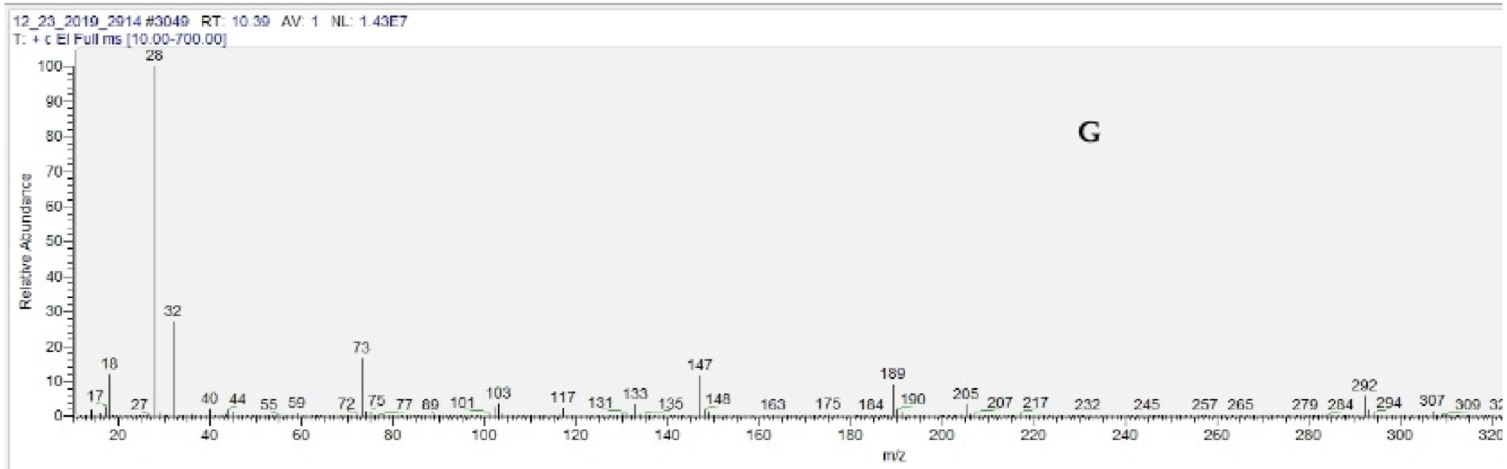

Figure S2. **A**: The mass spectrum of silylated liquid sample after glycerol hydrogenolysis reaction; **B**: The mass spectrum of TMS-Gly; **C**: The mass spectrum of TMS-EG; **D**: The mass spectrum of TMS-PG; **E**: The mass spectrum of TMS-LA; **F**: The mass spectrum of TMS-BD; **G**: The mass spectrum of TMS-GA.

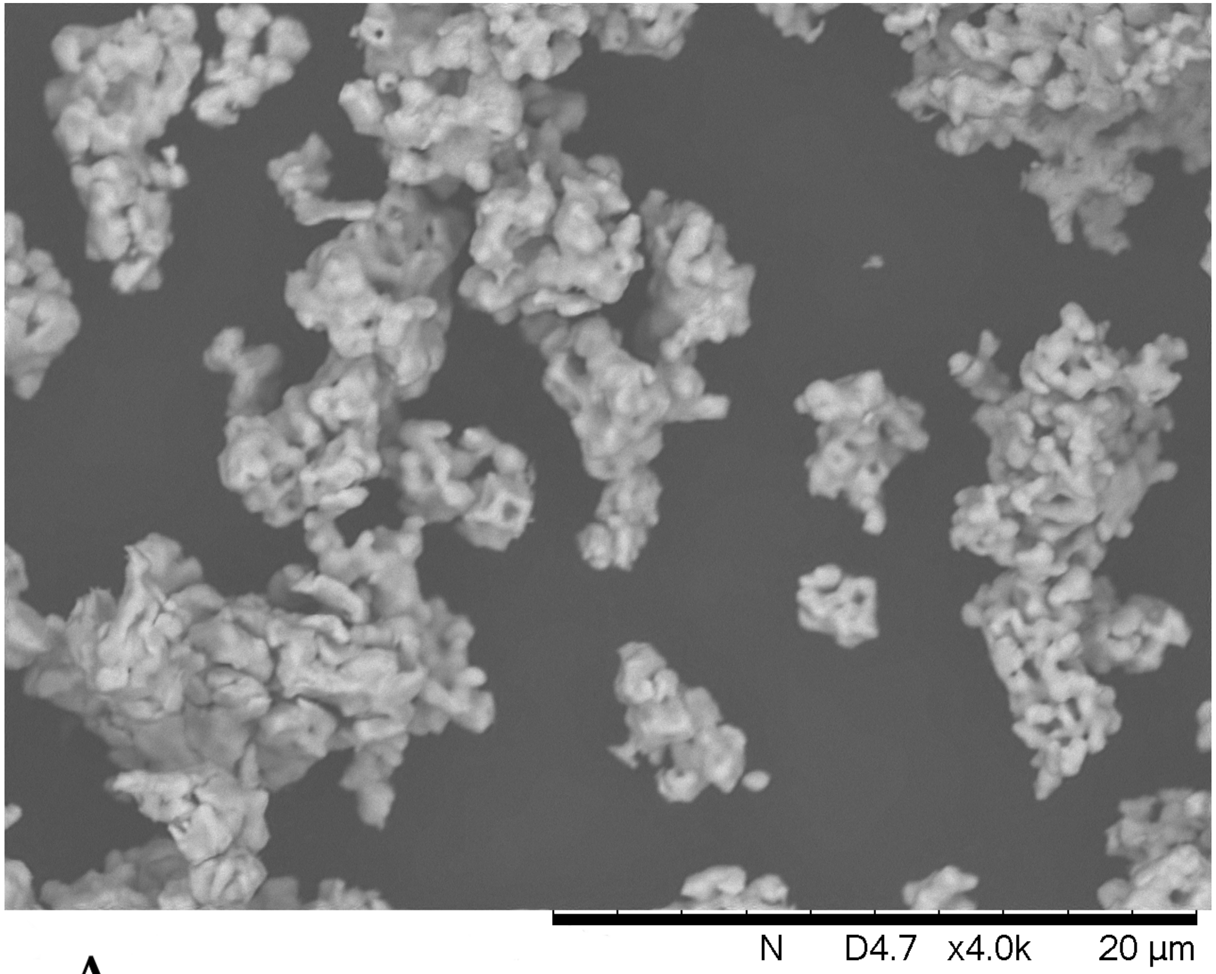

A

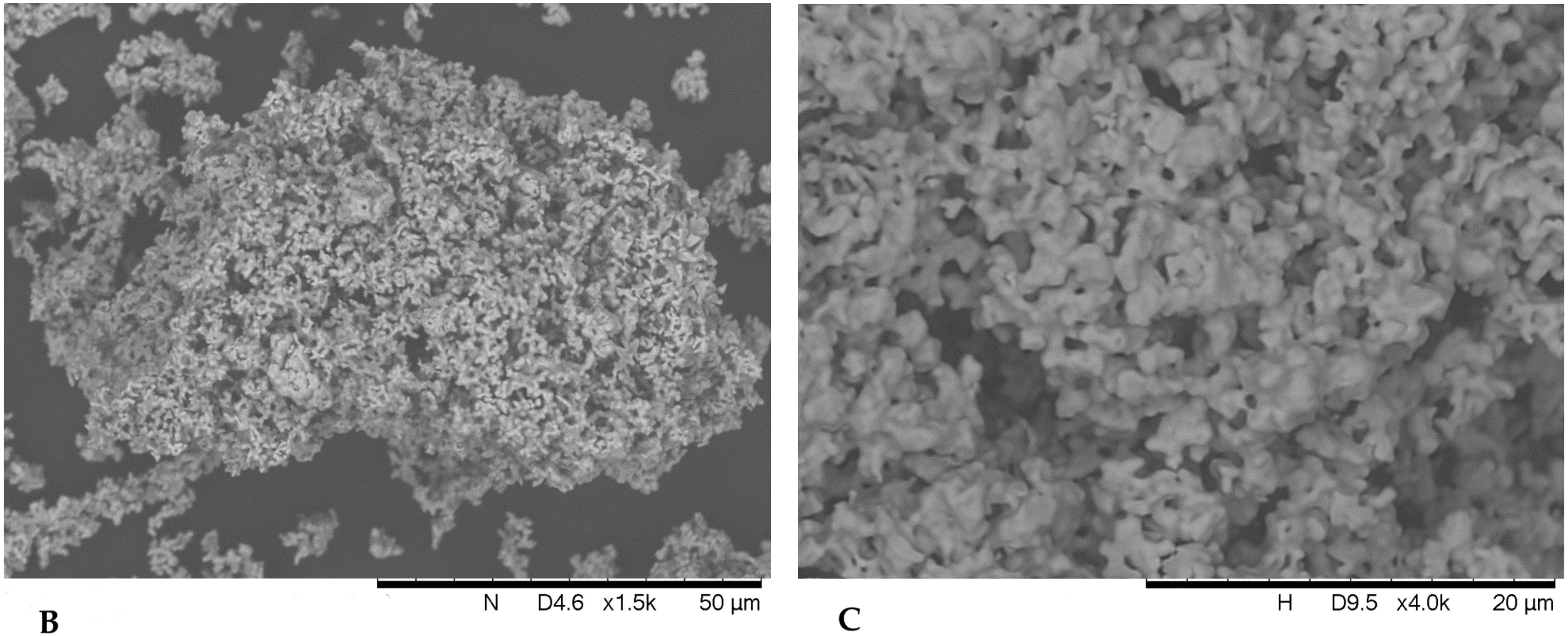

B C

Figure S3. The SEM microphotographs of the copper catalysts generated *in situ* in the reaction medium during hydrogenolysis of glycerol. **A, B:** Cu-5; **C**: Cu-15.

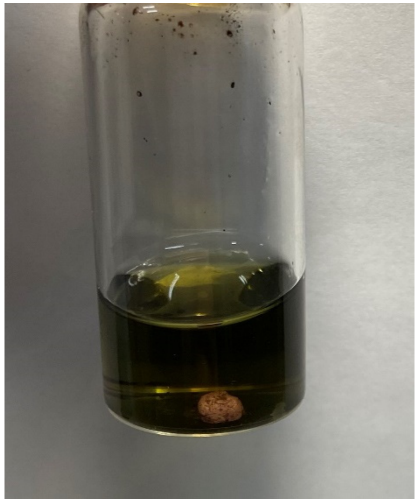

Figure S4. The appearance of the catalyst after 60 h of glycerol hydrogenolysis.

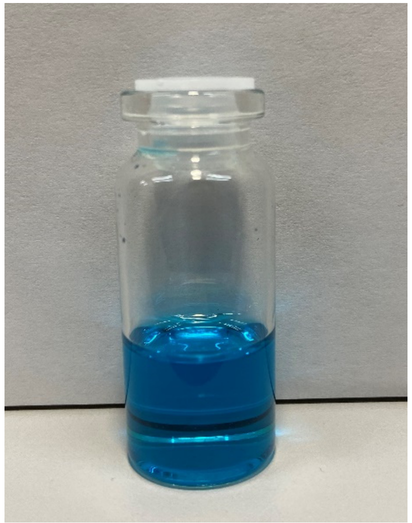

Figure S5. Cu(OAc)$_2$ in a water–glycerol solution prior to adding potassium hydroxide.
